# CD7‐targeting pro‐apoptotic extracellular vesicles: A novel approach for T‐cell haematological malignancy therapy

**DOI:** 10.1002/jev2.70025

**Published:** 2024-12-16

**Authors:** Bei Zhang, Jianqiang Chen, Jiming Chen, Yingying Shen, Yinghu Chen, Shibo Wang, Chengyan Zhang, Yuzhou He, Huajun Feng, Jiaoli Wang, Zhijian Cai

**Affiliations:** ^1^ Department of Orthopaedics of the Second Affiliated Hospital and Institute of Immunology Zhejiang University School of Medicine Hangzhou China; ^2^ Department of Blood Transfusion The First Affiliated Hospital, Zhejiang University School of Medicine Hangzhou China; ^3^ Key Laboratory of Functional and Clinical Translational Medicine, Fujian province university Xiamen Medical College Xiamen China; ^4^ Institute of Respiratory Diseases Xiamen Medical College Xiamen China; ^5^ Organiod platform of medical laboratory science Xiamen medical college Xiamen China; ^6^ Laboratory of Cancer Biology, Key Lab of Biotherapy in Zhejiang, Cancer Center of Zhejiang University, Sir Run Run Shaw Hospital Medical School of Zhejiang University Hangzhou China; ^7^ Department of Infectious Disease, Children’s Hospital, Zhejiang University School of Medicine, National Clinical Research Center for Child Health National Children’s Regional Medical Center Hangzhou China; ^8^ Department of Medical Oncology, Sir Run Run Shaw Hospital Zhejiang University School of Medicine Hangzhou China; ^9^ Department of Emergency The Second Affiliated Hospital of Zhejiang Chinese Medical University Hangzhou China; ^10^ Ecological‐Environment & Health College Zhejiang A & F University, Hangzhou Zhejiang China; ^11^ Affiliated Hangzhou First People's Hospital School of Medicine, Westlake University Hangzhou China

**Keywords:** anti‐CD7 single‐chain variable fragment, Bcl2 siRNA, cytochrome C, extracellular vesicles, T‐cell malignancies

## Abstract

T‐cell haematological malignancies progress rapidly and have a high mortality rate and effective treatments are still lacking. Here, we developed a drug delivery system utilizing 293T cell‐derived extracellular vesicles (EVs) modified with an anti‐CD7 single‐chain variable fragment (αCD7/EVs). Given the challenges of chemotherapy resistance in patients with T‐cell malignancy, we selected cytochrome C (CytC) and *Bcl2* siRNA (*siBcl2*) as therapeutic agents and loaded them into αCD7/EVs (αCD7/EVs/CytC/*siBcl2*). We found that αCD7/EVs efficiently targeted and were internalized by human T‐ALL Molt‐4 cells. In addition, the interaction between αCD7 and CD7 switched the EV entry pathway in Molt‐4 cells from macropinocytosis‐dependent endocytosis to clathrin‐mediated endocytosis, thereby reducing EV‐lysosome colocalization, ultimately improving CytC delivery efficiency and increasing the cytotoxicity of nascent EVs from EV‐treated Molt‐4 cells. Notably, αCD7/EVs/CytC/*siBcl2* demonstrated similar efficacy against both Molt‐4 and chemotherapy‐resistant Molt‐4 cells (CR‐Molt‐4). Furthermore, αCD7/EVs/CytC/*siBcl2* exhibited high safety, low immunogenicity and minimal impact on human T cells. Therefore, αCD7/EVs/CytC/*siBcl2* are promising therapeutic approaches for treating CD7^+^ T‐cell malignancies.

## 1 INTRODUCTION

T‐cell malignancies, including T‐cell lymphomas and T‐cell leukaemia, are highly aggressive and rapidly progressing tumours known to occur in paediatric (10%∼15%) and adult patients (20%∼25%) (Teachey & O'Connor, 2020; Touzart et al., 2021; Winter et al., 2018; Wu et al., 2023). The prognosis of T‐cell malignancies is generally poor, particularly in patients who have relapsed or are refractory, with a 5‐year overall survival rate of less than 20% (Hu et al., 2022; Marks & Rowntree, 2017). Intensive chemotherapy and hematopoietic stem cell transplantation are the primary curative treatment options for patients with T‐cell malignancies. However, these treatments can result in increased treatment‐related complications and mortality risks, including chemotherapy resistance, graft‐versus‐host disease, and systemic infection (Saad et al., 2020; Teachey & Pui, 2019). Chimeric antigen receptor (CAR)‐T‐cell immunotherapy has developed rapidly in recent decades (Hu et al., 2022; MacKay et al., 2020). CD7 is a transmembrane glycoprotein expressed in more than 90% of T‐cell acute lymphoblastic leukaemia (T‐ALL) and T‐cell lymphomas, making it an attractive immunotherapy target for T‐cell malignancies (Dai et al., 2022; Freiwan et al., 2022; Velasquez & Mamonkin, 2022). However, CD7 molecules are also expressed in CAR‐T cells and most normal T cells (Freiwan et al., 2022; Velasquez & Mamonkin, 2022), leading to compromised therapeutic effects because of CD7‐targeting CAR‐T cell fratricide (Kalluri & LeBleu, 2020) and graft‐versus‐host disease. In addition, cytokine storm syndrome is a substantial challenge of CAR‐T‐cell therapy. Therefore, there is an urgent need to develop innovative therapies for T‐cell malignancies.

Extracellular vesicles (EVs) are a group of heterogeneous nanoscale membrane‐structured particles actively released by various types of cells (Kalluri & LeBleu, 2020; van Niel et al., 2018). EVs can transfer diverse bioactive cargos, including nucleic acids, proteins, lipids, and metabolites, on their surface or inside their lumen (Kalluri & LeBleu, 2020; Maacha et al., 2019; van Niel et al., 2018). Therefore, the secretion of EVs has emerged as an essential mechanism of intracellular communication (Marar et al., 2021). In addition, EVs exhibit many favourable properties, such as physiochemical stability, good biocompatibility, low immunogenicity, biodegradability, and improved permeability of biological barriers (Escudé Martinez de Castilla et al., 2021; Tang et al., 2020; Zhang, Zhang, et al., 2021). As a result, they are considered excellent natural drug delivery and nanotherapeutic platforms and are widely used in the diagnosis and treatment of various diseases (Escudé Martinez de Castilla et al., 2021; Tang et al., 2020; Zhang, Zhang, et al., 2021; Zhang et al., 2024). EVs have great advantages, especially in the delivery of therapeutic RNA (de Voogt et al., 2021; Murphy et al., 2021). Recent efforts have emphasized engineering EVs to improve their targeting capability, loading efficiency and therapeutic effect (Dooley et al., 2021; Zhang et al., 2021). There are two main types of optimization techniques: (1) genetic or metabolic reprogramming of donor cells to produce engineered vesicles and (2) direct modification of purified EVs by multiple approaches (de Abreu et al., 2020; Fan et al., 2022; Richter et al., 2021). It has been reported that HEK293 cell‐derived EVs with high expression levels of CD40L, gp350, and pp65 can increase the antigen‐presenting ability to chronic lymphocytic leukaemia cells and stimulate the activation of CD4^+^ and CD8^+^ T cells specific to gp350 and pp65 (Gärtner et al., 2019). Our previous study demonstrated that gp350‐anchored red blood cell‐derived EVs loaded with doxorubicin (Dox) have a therapeutic effect on B‐cell malignancies (Xiu et al., 2022). On the basis of the promising results of recent studies, we speculate that engineered EV drug delivery systems should also have potential for T‐cell malignancy therapy.

The regulation of cell death as a target of anticancer drug development has attracted increasing attention (Tong et al., 2022). However, cancer cells, including haematological malignancies, gradually evolve and develop multidrug resistance during chemotherapy, which dramatically impacts the effectiveness of cancer treatment (Cui et al., 2018; Müller et al., 2022; Wu et al., 2023). Cytochrome C (CytC), an impermeable mitochondrial peripheral membrane protein, initiates intrinsic apoptosis (Yang et al., 2017). The critical step in intrinsic apoptosis is mitochondrial outer membrane permeabilization (MOMP), which promotes the release of CytC into the cytosol and the formation of apoptosomes (CytC, Apaf1 and the pro‐caspase 9 complex) (Bock & Tait, 2020; Dorstyn et al., 2018; Ow et al., 2008). However, antiapoptotic members of the Bcl2 family, such as Bcl2, Bcl‐XL and Bcl‐W, can prevent MOMP (Galluzzi et al., 2018). Nanoparticles of CytC combined with cardiolipin can penetrate both the cell and mitochondrial membranes, leading to lipid peroxidation reactions that induce the apoptosis of drug‐sensitive and Dox‐resistant cancer cells (Vladimirov et al., 2017). In addition, a Bcl2 inhibitor, venetoclax, has been demonstrated to be a precision medicine for myeloid leukaemia and to enhance clinical activity when combined with standard or novel drugs (Döhner et al., 2021; Hallek et al., 2018). Consequently, we assume that the application of EVs can deliver CytC to T‐cell malignancy and further promote apoptosis when combined with *Bcl2* siRNA (*siBcl2*).

Here, we designed an EV‐based drug delivery platform for CD7^+^ T‐cell malignancy treatment comprised of 293T cell‐derived EVs with high anti‐CD7 single‐chain variable fragments (αCD7/EVs) and potential therapeutic agents: CytC and *siBcl2* (αCD7/EVs/CytC/*siBcl2*). αCD7/EVs had a significant targeting effect on CD7^+^ T‐cell malignancy in vitro and in vivo because of the specific binding of αCD7 to CD7 molecules. When loaded with CytC and cholesterol‐modified single‐stranded *siBcl2*, αCD7/EVs/CytC/*siBcl2* demonstrated a robust capacity to induce apoptosis in human T‐ALL Molt‐4 cells and chemotherapy‐resistant Molt‐4 cells (CR‐Molt‐4), thereby alleviating T‐ALL regardless of chemotherapy resistance. Notably, this innovative therapeutic platform exhibited very low antigenicity, high safety and negligible effects on healthy T cells in mouse models. Consequently, αCD7/EVs/CytC/*Bcl2* represent a promising therapeutic reagent for CD7^+^ T‐cell malignancy.

## 2 RESULTS

### 2.1 Anchorage of αCD7 on EVs promotes EV uptake by CD7^+^ T‐cell malignancy

**FIGURE 1:**
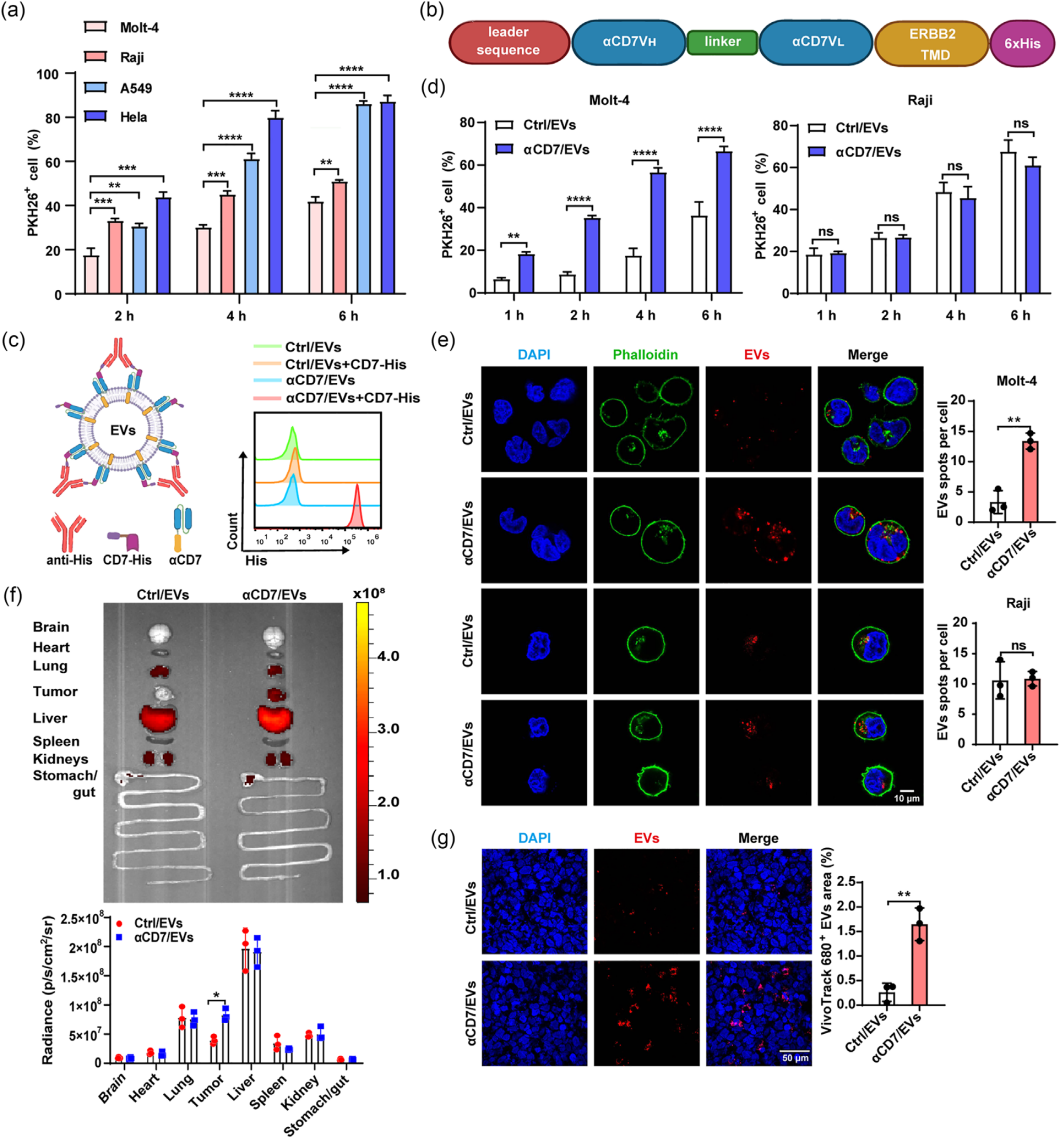
Anchorage of αCD7 on EVs promotes EV uptake by CD7^+^ T‐cell malignancies. (a) FACS analysis of the uptake of 1 µg of PKH26‐labelled 293T/EVs by Molt‐4, Raji, the HeLa human cervical cancer cell line and the A549 human non‐small cell lung cancer cell line for the indicated times. (b) Schematic diagram of the αCD7 structure. (c) The schematic diagram for detecting the αCD7 level on the EV surface (left). FACS analysis of αCD7 levels on the EV surface (right). (d) FACS quantification of the uptake of 1 µg of PKH26‐labelled EVs (Ctrl/EVs and αCD7/EVs ≈ 1.85 × 10^9^ particles) by Molt‐4 and Raji cells for the indicated times. (e) Representative confocal images (left) and quantification (right) of EV (red) uptake by Molt‐4 and Raji cells (green) for 4 h. Scale bars, 10 µm. Each dot indicates the number of EV spots per cell. (f) and (g) Molt‐4 tumour‐bearing NOD/SCID/*Il2rg* ^−/−^ (NSG) mice received an i.v. injection of 100 µg (≈ 1.79 × 10^11^ particles) VivoTrack 680‐labelled Ctrl/EVs or αCD7/EVs and were assessed after 24 h. (f) Representative IVIS images (top) and quantification (bottom) of EV uptake in the indicated organs and tumours from these mice. (g) Representative confocal images (left) and quantification (right) of VivoTrack 680‐labelled EVs in Molt‐4 tumour sections from these mice. Scale bars, 50 µm. Each point represents a field of view. The data are representative of three independent experiments (*n* = 3). The error bars represent the means ± SDs (ns, not significant, *p < 0.05, **p < 0.01, ***p < 0.001, ****p < 0.0001, unpaired Student's *t* test in [a]—[g]). EVs, extracellular vesicles; FACS, fluorescence‐activated cell sorting; i.v., intravenous; IVIS, in vivo imaging system.

EVs have shown great potential as a drug delivery system (Escudé Martinez de Castilla et al., 2021; Herrmann et al., 2021; Zhang, Jain, et al., 2021), prompting us to explore their application in treating CD7^+^ T‐cell malignancies. However, our study revealed that 293T‐derived EVs (293T/EVs) had a lower uptake by Molt‐4 cells compared to that of several common adherent and suspended tumour cell lines (Figure 1a). Similarly, the infiltration of 293T/EVs in Molt‐4 tumours was minimal (Figure S1a). Given the high expression levels of CD7 on the Molt‐4 cell surface (Figure S1b), we designed a lentiviral plasmid to prepare engineered EVs with enhanced targeting and uptake in Molt‐4 cells. This plasmid encodes αCD7 followed by a transmembrane domain (TMD) from the human *ERBB2* gene, allowing for effective anchoring of αCD7 on the cell membrane (Figure 1b). After lentiviral transduction, we generated 293T cells stably expressing αCD7 and isolated EVs from their culture supernatant. Coincubation with the purified CD7 protein (CD7‐His) confirmed the overexpression of αCD7 on the surface of both the cells and their EVs (αCD7/EVs) but not on the EVs from 293T cells with mock transfection (Ctrl/EVs) (Figure 1c and Figure S1c). The results also reflected the high affinity of αCD7 for CD7 (Figure 1c). Electron microscopy (EM) revealed that αCD7/EVs exhibited the classic ‘cup‐like’ shape characteristic of EVs (Figure S1d), with a diameter of approximately 200 nm, as detected by nanoparticle tracking analysis (NTA) (Figure S1e). EV markers, such as Tsg101, Alix and CD9, and the negative marker GRP94 were analyzed by western blotting (WB) (Figure S1f). All these characteristics were comparable to those of Ctrl/EVs. Additionally, silver staining revealed similar total protein expression between Ctrl/EVs and αCD7/EVs (Figure S1g). These results suggest that the overexpression of αCD7 did not essentially alter the EV characteristics.

To investigate the potential of αCD7/EVs to enhance uptake by CD7^+^ haematological malignancies, CD7^+^ Molt‐4 and CD7^−^ human Raji Burkitt's lymphoma cells were incubated with PKH26‐labelled Ctrl/EVs and αCD7/EVs for the indicated times. After three rounds of ultracentrifugation to remove excess dye, we confirmed that this method was sufficient to eliminate free dye, as indicated by the similar fluorescence intensity in the supernatant after the third ultracentrifugation and the negative control (PBS) (Figure S1h). Additionally, there was no significant difference in PKH26 fluorescence intensity between equal amounts of αCD7/EVs subjected to density gradient ultracentrifugation and those subjected to three rounds of ultracentrifugation washing (Figure S1i). Therefore, ultracentrifugation was used for dye removal in subsequent experiments, with a staining efficiency of 77%, as determined by nanoflow cytometry (Figure S1j). In addition, PKH26 staining did not affect EV morphology or particle size (Figure S1k,l). NTA revealed similar particle numbers per unit mass (µg) for Ctrl/EVs and αCD7/EVs, with a linear correlation between protein mass and particle number (Figure S1m). We then used the mass‒particle number standard curve to quantify the EV particles used in subsequent experiments. Fluorescence‐activated cell sorting (FACS) and confocal imaging revealed that CD7^+^ Molt‐4 internalized more αCD7/EVs than did Ctrl/EVs (Figure 1d,e), whereas no significant difference in the uptake of these EVs by CD7^−^ Raji cells was detected (Figure 1d,e). Blocking αCD7 on αCD7/EVs with CD7‐His abrogated the enhanced uptake by Molt‐4 cells (Figure S1n). Moreover, compared with wild‐type Raji cells, CD7‐overexpressing Raji (over‐Raji) cells exhibited increased uptake of αCD7/EVs (Figure S1o,p), indicating that the increased uptake of αCD7/EVs was dependent on the αCD7‐CD7 interaction.

To evaluate the ability of αCD7/EVs to target CD7+ tumours in vivo, VivoTrack 680‐labelled Ctrl/EVs and αCD7/EVs were intravenously injected in Molt‐4/Raji subcutaneous (s.c.) tumour‐bearing mice. Although αCD7/EVs were still predominantly distributed in the liver, they accumulated more in Molt‐4 tumours than did Ctrl/EVs (Figure 1f). Consistently, confocal imaging of tumour tissue from the mice receiving the αCD7/EVs revealed a larger fluorescence area than that of the control (Figure 1 g). However, no significant differences were observed in Raji tumours between the two EV types (Figure S1q,r). These results confirm that αCD7 modification enhanced EV targeting and internalization in CD7^+^ T‐cell malignancies.

### 2.2 αCD7/EVs loaded with CytC effectively inhibit CD7^+^ T‐cell malignancies

Next, we assessed the ability of αCD7/EVs to deliver therapeutic agents to CD7^+^ T‐cell malignancies. Since CytC can initiate intrinsic apoptosis but cannot independently penetrate the cell membrane, we hypothesized that αCD7/EVs loaded with CytC could facilitate its transport into Molt‐4 cells, thereby inducing apoptosis. Electroporation is a common and powerful technique for loading functional molecules in EVs (Lennaárd et al., 2021; Nasiri Kenari et al., 2020). Therefore, CytC was loaded into Ctrl/EVs and αCD7/EVs by electroporation (Ctrl/EVs/CytC and αCD7/EVs/CytC). Free CytC was removed by density gradient ultracentrifugation, with the majority of αCD7/EVs/CytC concentrated in the third fraction. Owing to EV aggregation during electroporation, a small amount was also detected in the ninth fraction (Figure S2a). We then combined these fractions for further experiments. Gray value analysis demonstrated that the CytC loading efficiency by ultracentrifugation was comparable to that by density gradient ultracentrifugation (Figure S2b), validating the use of ultracentrifugation for removing free CytC in subsequent experiments.

**FIGURE 2:**
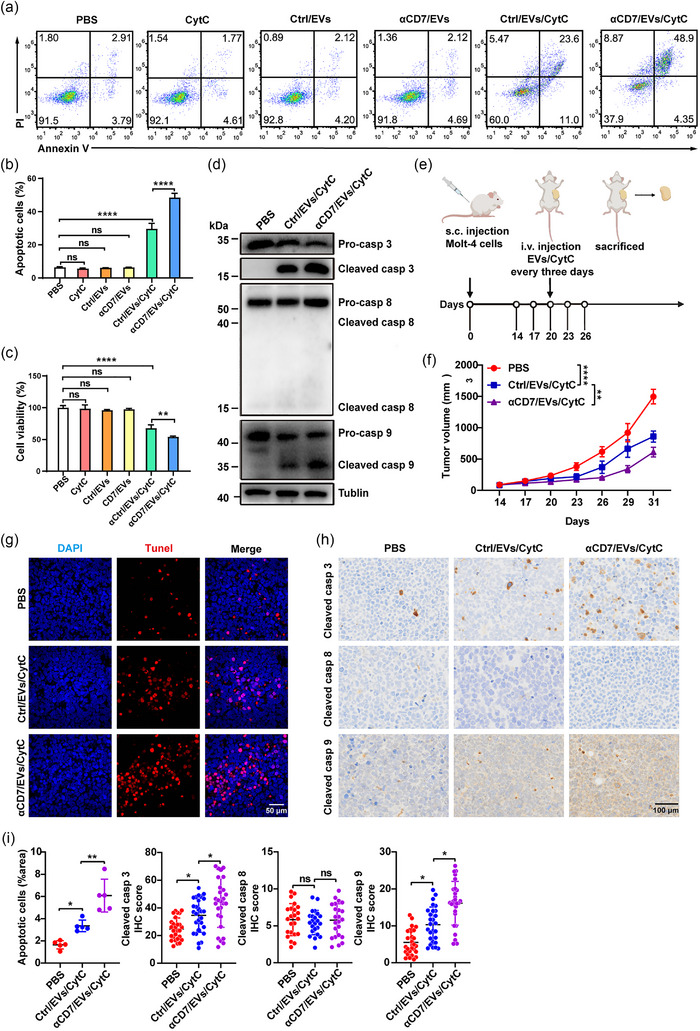
αCD7/EVs loaded with CytC effectively inhibit CD7^+^ T‐cell malignancies. (a)–(c) Molt‐4 cells were incubated with PBS, 10 µg of CytC, or 1.79 × 10^10^ particles of Ctrl/EVs, αCD7/EVs, Ctrl/EVs/CytC, or αCD7/EVs/CytC for 48 h (*n* = 3). (a) FACS analysis of apoptotic cells (%). (b) Quantification of apoptotic cells (%). (c) CCK8 analysis of the viability of these cells. (d) WB analysis of the protein markers of apoptosis in these cells. (e)–(i) Molt‐4 tumour‐bearing NSG mice were intravenously injected with 100 µg (≈ 1.79 × 10^11^ particles) of Ctrl/EVs/CytC or αCD7/EVs/CytC every 3 days for a total of 5 times (*n* = 5). (e) Schematic diagram of mouse tumour model construction and treatment protocols. (f) Tumour progression was evaluated on the basis of tumour size. (g) Representative images of TUNEL immunofluorescence staining of Molt‐4 tumour tissues from these mice. Scale bars, 50 µm. (h) Representative IHC staining images of cleaved caspase 3, 8 and 9 in Molt‐4 tumour tissues from the mice. Scale bars, 100 µm. (i) Quantification of apoptotic cells per reported area in (g) and cleaved caspase 3, 8 and 9 IHC staining in (h). Each dot indicates a randomly acquired image (*n* = 5 in (f), *n* = 23–27 in (g). The data are representative of three independent experiments. The error bars represent the means ± SDs (ns, not significant, *p < 0.05, **p < 0.01, ****p < 0.0001, one‐way ANOVA followed by Tukey's test in [b]–[i]). CytC, cytochrome C; EVs, extracellular vesicles; IHC, immunohistochemistry.

To confirm the incorporation of CytC into the EVs, we digested the EVs with proteinase k, which degraded surface proteins such as CD9 but did not affect internal proteins such as Alix, Tsg101, or CytC, indicating that CytC was indeed successfully loaded into the EVs (Figure S2c). WB revealed that the CytC loading efficiency was similar between Ctrl/EVs/CytC and αCD7/EVs/CytC under identical electroporation conditions. Furthermore, through the mass‒gray value standard curve of CytC, we calculated that approximately 1.17 µg of CytC was loaded into 10 µg of EVs (11.7% loading efficiency) (Figure S2d). As expected, both αCD7/EVs/CytC and Ctrl/EVs/CytC were able to transport CytC into Molt‐4 cells, but more CytC was detected in αCD7/EVs/CytC‐treated cells (Figure S2e). It was subsequently observed that αCD7/EVs/CytC treatment induced prominent apoptosis and affected the viability of Molt‐4 cells, whereas neither αCD7/EVs nor CytC alone had these effects (Figure 2a–c). Although Ctrl/EVs/CytC also notably induced Molt‐4 cell apoptosis and reduced their viability, the effects were considerably weaker (Figure 2a–c). Consistent with the mechanism by which CytC is involved in intrinsic apoptosis, αCD7/EVs/CytC were capable of eliciting the cleavage of initiator caspase 9 and executioner caspase 3 in Molt‐4 cells, which was more potent than that elicited by Ctrl/EVs/CytC (Figure 2d). However, neither treatment caused the cleavage of caspase 8 (initiator of extrinsic apoptosis) (Figure 2d).

Next, we evaluated the antitumour effects of Ctrl/EVs/CytC and αCD7/EVs/CytC in vivo using a Molt‐4 tumour‐bearing mouse model. After intravenous injection (Figure 2e), both αCD7/EVs/CytC and Ctrl/EVs/CytC significantly suppressed the growth of Molt‐4 tumours, but αCD7/EVs/CytC exhibited a stronger targeting (Figure S2f) and inhibitory effect (Figure 2f). This was accompanied by increased apoptosis in tumour tissues, along with elevated levels of cleaved caspase 3 and caspase 9 but not cleaved caspase 8 (Figure 2 g–i). Although both EVs markedly inhibited Raji tumour growth, the inhibitory effects of Ctrl/EVs/CytC and αCD7/EVs/CytC were comparable (Figure S2g). To rule out the possibility that the observed inhibition was due to the EVs themselves or to CytC alone, we performed control experiments using Ctrl/EVs, αCD7/EVs, and CytC. As expected, neither type of EVs prevented Molt‐4 tumour growth, and even if the amount of CytC used was 8.5 times greater than that carried by EVs/CytC, CytC alone could also not inhibit Molt‐4 tumour development (Figure S2h). In conclusion, these results demonstrate that αCD7/EVs/CytC have the potential to treat CD7^+^ T‐cell malignancies by inducing intrinsic apoptosis through CytC delivery.

### 2.3 αCD7 modification alters EV endocytosis and facilitates offspring EV cytotoxicity

**FIGURE 3:**
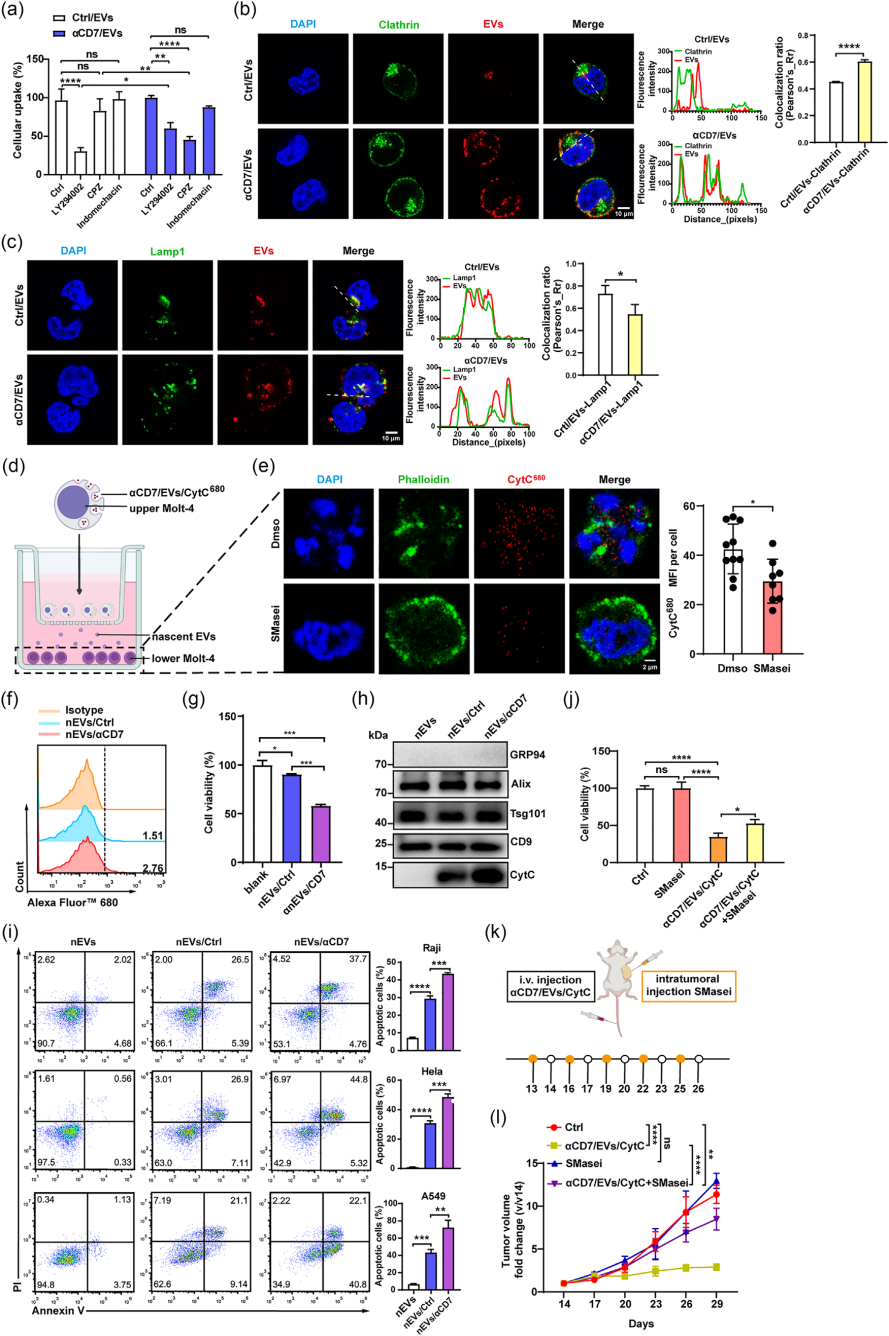
αCD7 modification alters EV endocytosis and facilitates nEV cytotoxicity. (a) FACS analysis of the 4 h uptake of 1 µg (≈ 1.85 × 10^9^ particles) of PKH26‐labelled Ctrl/EVs or αCD7/EVs by Molt‐4 cells pretreated with the indicated endocytosis inhibitors for 1 h (*n* = 3). (b) Representative confocal images (left) of the colocalization of PKH26‐labelled EVs with clathrin (green). Scale bars, 10 µm. Fluorescence profile analysis and colocalization ratio analysis (right) of PKH26‐labelled EVs and clathrin (green) (*n* = 3). (c) Representative confocal images (left) of the colocalization of PKH26‐labelled EVs with Lamp1 (green). Scale bars, 10 µm. Fluorescence profile analysis and colocalization ratio analysis (right) of PKH26‐labelled EVs and Lamp1 (green) (*n* = 3). (d) Schematic diagram of CytC^680+^ nEV uptake by Molt‐4 cells in the lower chamber. (e) Representative confocal images (left) and quantification (right) of CytC^680^ (red) in Molt‐4 cells (green) in the lower chamber (*n* = 3). Scale bars, 2 µm. Each dot indicates the mean fluorescence intensity of CytC^680^ per cell. (f) FACS analysis of nEVs/Ctrl^680^ and nEVs/αCD7^680^ isolated from an equal number of Molt‐4 cells after treatment with 20 µg (≈ 3.58 × 10^10^ particles) of Ctrl/EVs/CytC^680^ or 10 µg (≈ 1.79 × 10^10^ particles) of αCD7/EVs/CytC^680^ for 12 h. (g) Molt‐4 cells were treated with nEVs/Ctrl^680^ and nEVs/αCD7^680^ isolated from an equal number of Molt‐4 cells for 48 h. The viability of these cells was determined by a CCK8 assay (*n* = 3). (h) WB analysis of CytC in nEVs (from Molt‐4 cells treated with PBS), nEVs/Ctrl and nEVs/αCD7. (i) FACS analysis (left) and quantification (right) of the proapoptotic effect of 10 µg of nEVs/αCD7 or nEVs/Ctrl on the indicated tumour cells (*n* = 3). (j) Molt‐4 cells were pretreated with 5 µM SMasei for 2 h, followed by treatment with αCD7/EVs/CytC for 48 h. The viability of these cells was determined by a CCK8 assay (*n* = 3). (k), Schematic diagram of the mouse tumour model construction and treatment protocol. (l) Tumour size of Molt‐4 tumour‐bearing NSG mice with intratumoural injection of 2.5 µg/g SMasei with or without i.v. injection of 100 µg αCD7/EVs/CytC (*n* = 5). The data are representative of three independent experiments. The error bars represent the means ± SDs (ns, not significant, *p < 0.05, **p < 0.01, ***p < 0.001, ****p < 0.0001; two‐way ANOVA followed by multiple comparison test in [a]; unpaired Student's *t* test in [b]—[e]; one‐way ANOVA followed by Tukey's test in [g]–[l]). CytC, cytochrome C; EVs, extracellular vesicles; FACS, fluorescence‐activated cell sorting; i.v., intravenous.

To explore whether αCD7 modification alters the EV internalization pathway in CD7^+^ T‐cell malignancies, we pretreated Molt‐4 cells with several specific endocytosis inhibitors, including the macropinocytosis inhibitor LY294002, the caveolae‐mediated endocytosis inhibitor indomethacin and the clathrin‐mediated endocytosis inhibitor chlorpromazine (CPZ). First, we confirmed that none of the inhibitors affected the viability of Molt‐4 cells (Figure S3a). When the cells were incubated with PKH26‐labelled EVs, LY294002 effectively inhibited the endocytosis of both types of EVs in Molt‐4 cells, with a greater inhibition of Ctrl/EVs (Figure 3a). Indomethacin did not affect the uptake of either type of EVs (Figure 3a). Interestingly, when Molt‐4 cells were pretreated with CPZ, the endocytosis of αCD7/EVs rather than Ctrl/EVs was significantly suppressed (Figure 3a). These observations indicated a shift from macropinocytosis‐dependent endocytosis to clathrin‐mediated endocytosis. Clathrin‐mediated endocytosis is crucial for the internalization of transmembrane receptors and their associated ligands (Kaksonen & Roux, 2018; Mettlen et al., 2018). As a transmembrane glycoprotein, CD7 binding to its specific ligand triggers receptor‐ligand complex internalization into cells (Zhang, Jain, et al., 2021). Thus, we speculated that the shift in EV endocytosis may be due to the binding of CD7 to αCD7. As expected, PKH26‐labelled αCD7/EVs colocalized with clathrin in Molt‐4 cells, whereas Ctrl/EVs did not (Figure 3b). To further confirm the above findings, αCD7 on the αCD7/EV surface was blocked by CD7‐His, and the endocytosis transition was eliminated (Figure 3b).

Next, we explored the intracellular transport of αCD7/EVs. In Molt‐4 cells, neither αCD7/EVs nor Ctrl/EVs colocalized with EEA1^+^ early endosomes, but both types of EVs eventually colocalized with CD63^+^ multivesicular bodies and Lamp1^+^ lysosomes (Figure 3c and Figure S3c). Despite greater uptake of αCD7/EVs by Molt‐4 cells, these EVs exhibited lower colocalization with Lamp1 than did Ctrl/EVs (Figure 3c), indicating that clathrin‐mediated endocytosis of αCD7/EVs inclined them to lysosomal escape. We hypothesized that this lysosomal escape mechanism could allow more CytC to avoid degradation and be encapsulated into nascent EVs (nEVs) secreted by EV/CytC‐treated Molt‐4 cells during EV biosynthesis, thereby contributing to cytotoxicity. To verify this, Alexa Fluor 680‐labelled CytC was loaded into αCD7/EVs (αCD7/EVs/CytC^680^) and incubated with Molt‐4 cells with or without an N‐SMase spiroepoxide inhibitor (SMasei) to inhibit nEV release. Although the SMasei effectively inhibited EV production (Figure S3d), it did not affect αCD7/EV uptake (Figure S3e). After the removal of residual αCD7/EVs/CytC^680^, the treated (upper Molt‐4) and untreated cells (lower Molt‐4) were placed in the upper and lower Transwell chambers, respectively (Figure 3d). As expected, fluorescence at 680 nm was detected in the lower Molt‐4 cells and decreased with SMasei treatment (Figure 3e, Figure S3f), accompanied by a reduction in apoptosis levels (Figure S3 g).

To investigate the impact of lysosomal escape on nEV toxicity, different doses of Ctrl/EVs/CytC^680^ and 10 µg of αCD7/EVs/CytC^680^ (≈ 1.79 × 10^10^ particles) were incubated with Molt‐4 cells. FACS revealed that a 2:1 ratio of Ctrl/EVs/CytC^680^ to αCD7/EVs/CytC^680^ resulted in equal CytC^680^ uptake (Figure S3h). Under these conditions, nEVs from αCD7/EVs/CytC^680^‐treated Molt‐4 cells (nEVs/αCD7^680^) contained higher CytC overall (Figure 3f) and exhibited greater cytotoxicity than nEVs from an equal number of Ctrl/EVs/CytC^680^‐treated cells (nEVs/Ctrl^680^) (Figure 3 g). Next, we collected a large number of nEVs and confirmed that all types of nEVs had a typical EV morphology with a diameter of approximately 170 nm (Figure S3i,j). CytC was observed in both types of nEVs, with higher levels found in nEVs/αCD7 (Figure 3h). Additionally, silver staining revealed similar total protein expression in nEVs, nEVs/Ctrl and nEVs/αCD7 (Figure S3k). Similarly, when incubated with Raji, HeLa and A549 cells, nEVs/αCD7 exhibited increased cytotoxicity (Figure 3i). However, since Ctrl/EVs/CytC and αCD7/EVs/CytC contained the same amount of CytC, their apoptotic effects on the above cells were comparable (Figure S3l). These results suggest that lysosomal escape facilitated the incorporation of more CytC into nEVs/αCD7, thereby amplifying their cytotoxic effects. To further confirm the amplification of nEV‐mediated cytotoxicity, Molt‐4 cells were pretreated with the SMasei. SMasei treatment significantly reduced the cytotoxicity mediated by αCD7/EVs/CytC (Figure 3j). Combination treatment with αCD7/EVs/CytC and SMasei markedly impaired the antitumour effect of αCD7/EVs/CytC in vivo (Figure 3k,l), and SMasei treatment was confirmed to reduce EV production from Molt‐4 tumours (Figure S3m). In addition, SMasei treatment did not affect the tissue distribution or tumour‐targeting ability of αCD7/EVs (Figure S3n). Taken together, these results suggest that αCD7/EVs/CytC enhanced cytotoxicity by promoting the production of CytC‐containing nEVs, which amplified the antitumour effect through lysosomal escape.

### 2.4 Bcl2 silencing enhances the therapeutic effects of αCD7/EVs/CytC on CD7^+^ T‐cell malignancies

MOMP initiates the activation of caspase 9, establishing a robust feedforward mechanism to amplify cell death, whereas Bcl2 inhibits MOMP (Green & Kroemer, 2004). Therefore, we hypothesized that inhibiting Bcl2 would promote MOMP and improve the ability of αCD7/EVs/CytC to treat CD7^+^ T‐cell malignancies. Given that cholesterol‐conjugated siRNA can efficiently load into EVs (Haraszti et al., 2018), we synthesized cholesterol‐conjugated FAM‐labelled *siBcl2* and incubated it with αCD7/EVs (αCD7/EVs/*siBcl2*) or αCD7/EVs/CytC (αCD7/EVs/CytC/*siBcl2*). Immunofluorescence staining revealed that *siBcl2* could be loaded onto EVs (Figure S4a). Quantification by the FAM intensity‒mass curve indicated that the optimal loading efficiency was achieved when the *siBcl2*‐to‐EV mass ratio was 1:4, with approximately 17.12 µg of *siBcl2* being loaded into 100 µg of EVs (Figure S4b). The gel electrophoresis results indicated that free *siBcl2* could shift to the positive electrode and was almost completely degraded after incubation with fetal bovine serum (FBS), whereas αCD7/EVs/s*iBcl2* migrated slowly and resisted FBS degradation, which was consistent with reports that cholesterol promotes RNA entry into EVs (Zhan et al., 2020) (Figure S4c).

**FIGURE 4:**
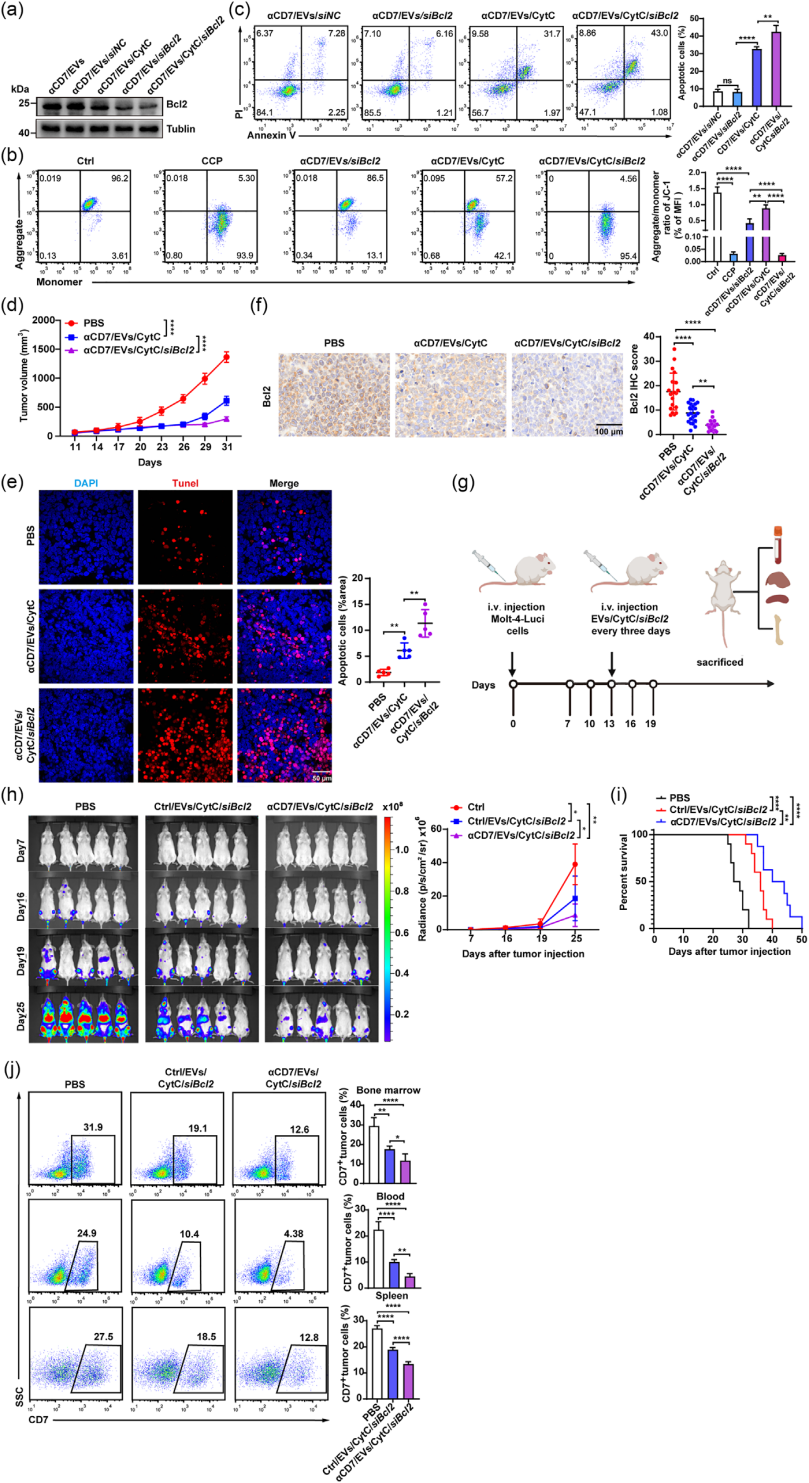
Bcl2 silencing enhances the therapeutic effects of αCD7/EVs/CytC on CD7^+^ T‐cell malignancy. (a)–(c) Molt‐4 cells were treated with 10 µg (≈ 1.79 × 10^10^ particles) of αCD7/EVs/*siNC*, αCD7/EVs/*siBcl2*, αCD7/EVs/CytC or αCD7/EVs/CytC/*siBcl2* for 48 h. (a) WB analysis of the Bcl2 protein in these cells. (b) FACS analysis (left) and quantification (right) of MOMP with JC‐1 dye. Mitochondrial membrane depolarization was demonstrated by the change in JC‐1 fluorescence from JC‐1 aggregates to JC‐1 monomers. CCP, a mitochondrial electron transport chain inhibitor, was used as a positive control. (c) FACS analysis (left) and quantification (right) of apoptosis in these cells (*n* = 3). (d)–(f) Molt‐4 tumour‐bearing NSG mice were intravenously injected with 100 µg (≈ 1.79 × 10^11^ particles) of αCD7/EVs/CytC or αCD7/EVs/CytC/*siBcl2* every 3 days for a total of 5 times (*n* = 5). (d) tumour progression was evaluated on the basis of tumour size. (e) Representative images (left) and quantification (right) of TUNEL immunofluorescence staining of Molt‐4 tumour tissues from these mice. Scale bars, 50 µm. Each dot indicates a randomly acquired image (*n* = 5). (f) Representative IHC images (left) and quantification (right) of the Bcl2 protein in Molt‐4 tumour tissues from these mice. Scale bars, 100 µm. Each dot indicates a randomly acquired image (*n* = 21). (g)–(j) NSG mice with T‐ALL were intravenously injected with 100 µg (≈ 1.79 × 10^11^ particles) Ctrl/EVs/CytC/*siBcl2* or αCD7/EVs/CytC/*siBcl2* every 3 days for a total of 5 times (*n* = 5). (g) Schematic diagram of T‐ALL mouse model construction, treatment protocol and analysis. (h) Representative IVIS images of T‐ALL progression (left) and quantification (right) of Molt‐4‐Luci signal intensity. (i) Survival curves of these mice. (j) FACS analysis (left) and quantification (right) of the infiltration of Molt‐4‐Luci cells in the peripheral blood, bone marrow and spleen. The data are representative of three independent experiments. The error bars represent the means ± SDs (ns, not significant, *p < 0.05, **p < 0.01, ***p < 0.001, ****p < 0.0001; one‐way ANOVA followed by Tukey's test in [b]–[j]; log‐rank test in [i]). CytC, cytochrome C; EVs, extracellular vesicles; FACS, fluorescence‐activated cell sorting; IHC, immunohistochemistry; IVIS, in vivo imaging system; MOMP, mitochondrial outer membrane permeabilization.

Molt‐4 cells treated with αCD7/EVs/*siBcl2* or αCD7/EVs/CytC/*siBcl2* presented a significant reduction in Bcl2 protein levels, whereas αCD7/EVs/*siNC* (loaded with negative control siRNA) treatment did not. Owing to CytC‐induced apoptosis, the αCD7/EVs/CytC group also presented decreased Bcl2 levels (Figure 4a). To evaluate the effect of *siBcl2* on MOMP, we used the aggregate/monomer ratio of JC‐1 dye to indicate mitochondrial membrane depolarization. Both the αCD7/EV/*siBcl2* and αCD7/EV/CytC treatments induced partial depolarization of the mitochondrial membrane, whereas the αCD7/EV/CytC/*siBcl2* treatment led to complete membrane potential loss (Figure 4b). This complete depolarization resulted in significantly greater levels of apoptosis in Molt‐4 cells than in αCD7/EVs/CytC‐treated cells. Although αCD7/EVs/*siBcl2* were able to reduce Bcl2 and cause MOMP, they seemed insufficient to induce apoptosis on their own (Figure 4c). Consistent with the above results, αCD7/EVs/CytC/*siBcl2* induced more caspase 3 and caspase 9 cleavage than did αCD7/EVs/CytC, whereas αCD7/EVs/*siBcl2* did not induce caspase cleavage. Moreover, none of the treatments caused the cleavage of caspase 8 (Figure S4d).

Next, we evaluated the therapeutic effect of αCD7/EVs/CytC/*siBcl2* on Molt‐4 tumours. First, we confirmed that loading *Bcl2* siRNA did not affect the ability of αCD7/EVs/CytC to target Molt‐4 tumours (Figure S4e). Consistent with the in vitro results, both αCD7/EVs/CytC and αCD7/EVs/CytC/*siBcl2* strongly inhibited Molt‐4 tumour growth, but αCD7/EVs/CytC/*siBcl2* exhibited significantly stronger inhibitory effects (Figure 4d). Moreover, tumours from mice treated with αCD7/EVs/CytC/*siBcl2* also contained more apoptotic cells and reduced Bcl2 protein levels (Figure 4e,f). Additionally, increased levels of cleaved caspase 3 and 9 but not cleaved caspase 8 were observed in tumour tissues from the αCD7/EVs/CytC/*siBcl2* group (Figure S4f).

Next, we evaluated the effects of αCD7/EVs/CytC/*siBcl2* on T‐ALL. To induce T‐ALL, we intravenously injected Molt‐4 cells stably expressing luciferase (Molt‐4‐Luci) into NSG mice and treated these mice with Ctrl/EVs/CytC/*siBcl2* or αCD7/EVs/CytC/*siBcl2* (Figure 4g). The bioluminescence signals from Molt‐4‐Luci cells spread throughout the bodies of T‐ALL mice as leukaemia progressed. However, treatment with αCD7/EVs/CytC/*siBcl2* significantly reduced the signals and prolonged the survival time of these mice (Figure 4 h,i). Numerous Molt‐4 cells accumulated in the bone marrow, peripheral blood and spleen of T‐ALL mice, but their accumulation was significantly inhibited by αCD7/EV/CytC/*siBcl2* treatment (Figure 4j). Histopathological (H&E) staining results further confirmed decreased infiltration of Molt‐4‐Luci cells into the liver following αCD7/EV/CytC/*siBcl2* treatment (Figure S4g). In addition, the severe weight loss induced by T‐ALL was significantly alleviated after treatment with αCD7/EVs/CytC/*siBcl2* (Figure S4h). Although all these phenotypes were also eased by Ctrl/EVs/CytC/*siBcl2*, those phenotypes were much weaker than those of αCD7/EVs/CytC/*siBcl2* (Figure 4h–j and Figure S4g,h). In conclusion, these results demonstrate that αCD7/EVs/CytC/*siBcl2* had excellent therapeutic effects on T‐ALL by enhancing antitumour effects through Bcl2 inhibition and subsequent MOMP activation.

### 2.5 αCD7/EVs/CytC/*siBcl2* effectively inhibit chemotherapy‐resistant CD7^+^ T‐cell malignancies

**FIGURE 5:**
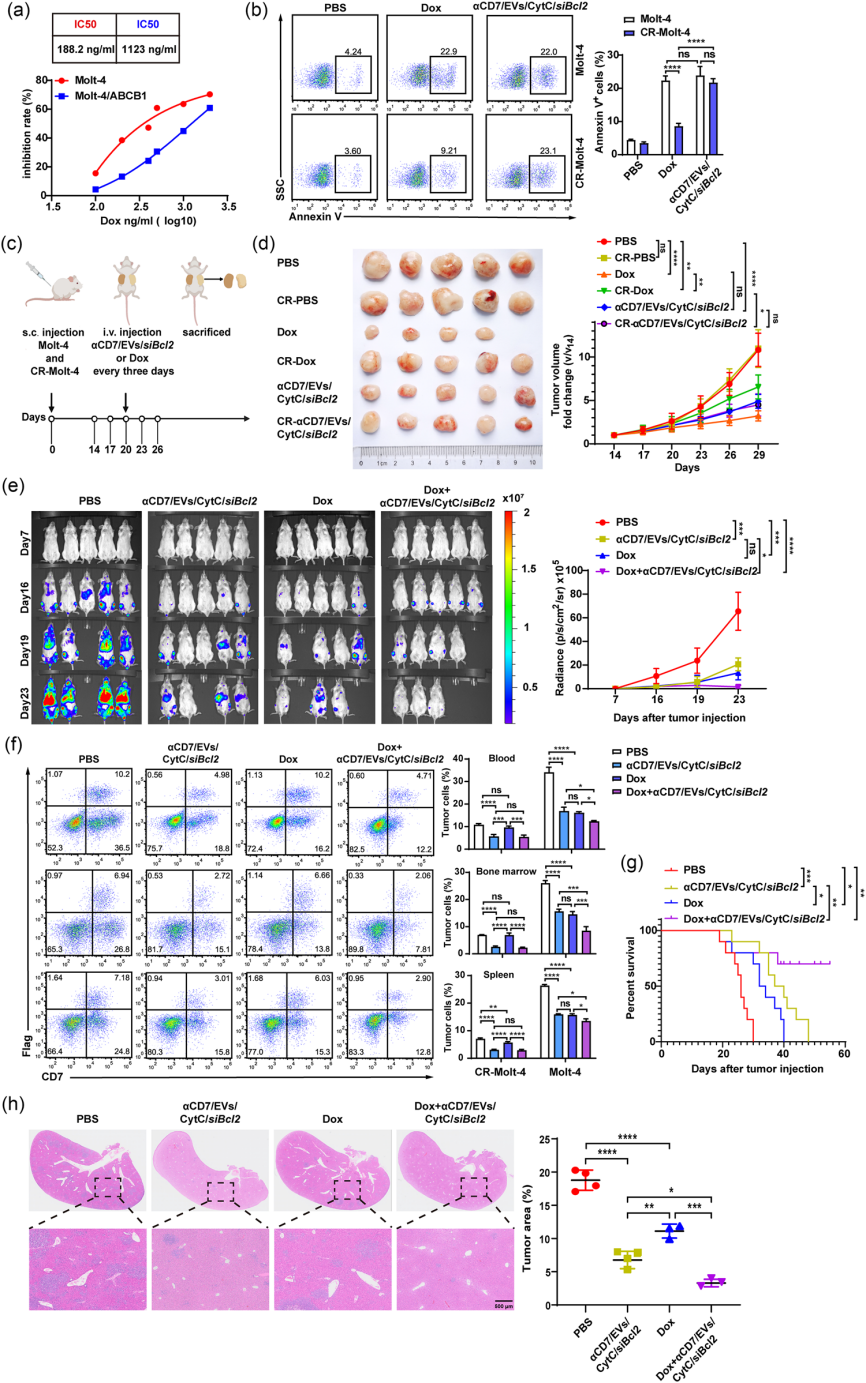
αCD7/EVs/CytC/*siBcl2* effectively inhibit chemotherapy‐resistant CD7^+^ T‐cell malignancies. (a) CCK8 analysis of the viability of Molt‐4 cells and CR‐Molt‐4 cells treated with the indicated concentrations of Dox. (b) FACS analysis (left) and quantification (right) of apoptosis in Molt‐4 cells and CR‐Molt‐4 cells treated with 10 µg (≈ 1.79 × 10^10^ particles) of αCD7/EVs/CytC/*siBcl2* or 100 ng/mL Dox for 24 h. (c) Schematic diagram of the mouse tumour model construction and treatment protocol. Tumour‐bearing NSG mice were intravenously injected with 100 µg (≈ 1.79 × 10^11^ particles) of αCD7/EVs/CytC/*siBcl2* or 2.5 µg/g Dox every three days for a total of 5 times (*n* = 5). (d) Tumour progression was evaluated on the basis of tumour size. (e)–(h) NSG mice with T‐ALL were intravenously injected with 100 µg (≈ 1.79 × 10^11^ particles) of αCD7/EVs/CytC/*siBcl2*, 2.5 µg/g Dox or 100 µg (≈ 1.79 × 10^11^ particles) of αCD7/EVs/CytC/*siBcl2* + 2.5 µg/g Dox every 3 days for a total of 5 times (*n* = 5). (e) Representative IVIS images (left) and quantification (right) of Molt‐4‐Luci signal intensity. (f) FACS analysis (left) and quantification (right) of the infiltration of Molt‐4‐Luci and CR‐Molt‐4 cells in peripheral blood, bone marrow and spleen (*n* = 3). (g) Survival curve of these mice. (h) Representative H&E staining images (left) and quantification (right) of the infiltration of T‐ALL in liver lobes (*n* = 4–3). The data are representative of three independent experiments. The error bars represent the means ± SDs (ns, not significant, *p < 0.05, **p < 0.01, ***p < 0.001, ****p < 0.0001; two‐way ANOVA followed by multiple comparisons test in [b]; one‐way ANOVA followed by Tukey's test in [d]–[h]; log‐rank test in [g]). CytC, cytochrome C; EVs, extracellular vesicles.

Although high‐dose chemotherapy has notably improved the prognosis for patients with T‐cell malignancies, relapsed patients, particularly adults and a small number of children, continue to have a poor prognosis, which is attributed primarily to the frequent development of drug resistance (García‐Hernández et al., 2023; Tian et al., 2020). As an ATP‐binding cassette (ABC) transporter member, ABC subfamily B member 1 (ABCB1) plays a critical role in multidrug resistance by facilitating chemotherapy drug efflux across cellular membranes. (Wu et al., 2021; Zhang et al., 2022). To evaluate the therapeutic potential of αCD7/EVs/CytC/*siBcl2* in chemotherapy‐resistant tumours, we established chemotherapy‐resistant Molt‐4 cells (CR‐Molt‐4 cells) by transfecting an ABCB1‐expressing lentivirus. WB analysis confirmed the high expression of flag‐ABCB1 in CR‐Molt‐4 cells (Figure S5a). Compared with parental Molt‐4 cells, CR‐Molt‐4 cells treated with different concentrations of Dox presented greater viability and a higher IC50, confirming their resistance (Figure 5a). In addition, CR‐Molt‐4 cells maintained a similar ability to take up EVs as their parental counterparts did (Figure S5b). Notably, when αCD7/EVs/CytC/*siBcl2* had the same effect as Dox on parental Molt‐4 cells, αCD7/EVs/CytC/*siBcl2* exhibited a superior efficacy in inducing the apoptosis of CR‐Molt‐4 cells (Figure 5b). Next, we used a subcutaneous tumour model to evaluate the therapeutic effects of αCD7/EVs/CytC/*siBcl2* in vivo (Figure 5c). While αCD7/EVs/CytC/*siBcl2* had the same inhibitory effect on Molt‐4 tumours as Dox did, it significantly outperformed Dox in suppressing the growth of CR‐Molt‐4 tumours (Figure 5d). This finding was consistent with the observed decreases in Bcl2 levels and increases in apoptosis in tumour tissues from αCD7/EV/CytC/*siBcl2*‐treated mice (Figure S5c,d).

To better mimic T‐ALL heterogeneity, we constructed a T‐ALL mouse model by mixing Molt‐4‐Luci with CR‐Molt‐4 cells at a 10:1 ratio. Both Dox and αCD7/EVs/CytC/*siBcl2* similarly reduced the bioluminescence signals from Molt‐4‐Luci cells. Moreover, combination treatment (Dox + αCD7/EVs/CytC/*siBcl2*) further intensified this reduction (Figure 5e). Analysis of CR‐Molt‐4 cell distribution in the bone marrow, peripheral blood and spleen revealed that both αCD7/EV/CytC*/siBcl2* treatment and combination therapy comparably decreased cell accumulation. However, Dox alone resulted in relatively high levels of CR‐Molt‐4 cells. For Molt‐4 cells, both treatments similarly inhibited cell accumulation, with the combination therapy having the strongest inhibitory effect (Figure 5f). These findings indicate that CR‐Molt‐4 cells in mixed T‐ALL were eliminated mainly by αCD7/EVs/CytC/*siBcl2* and that combined therapy further inhibited parental cells. Moreover, all the treatments prolonged the survival time of the T‐ALL mice, with the combination treatment inducing the greatest improvement (Figure 5 g). Histopathological results revealed that, compared with the αCD7/EV/CytC/*siBcl2*‐treated group, the Dox‐treated group exhibited greater infiltration of mixed tumour cells into the liver. Moreover, the combination therapy group displayed minimal infiltration (Figure 5h). In addition, T‐ALL‐induced weight loss was alleviated by αCD7/EV/CytC/s*iBcl2* treatment (Figure S5e). These findings suggest that αCD7/EVs/CytC/*siBcl2* has the potential to overcome the resistance of T‐ALL to chemotherapy and that the combination of αCD7/EVs/CytC/*siBcl2* with chemotherapy will further enhance their therapeutic efficacy by killing chemosensitive malignant cells more thoroughly.

### 2.6 αCD7/EVs/CytC/*siBcl2* have a high biological safety and low immunogenicity

**FIGURE 6:**
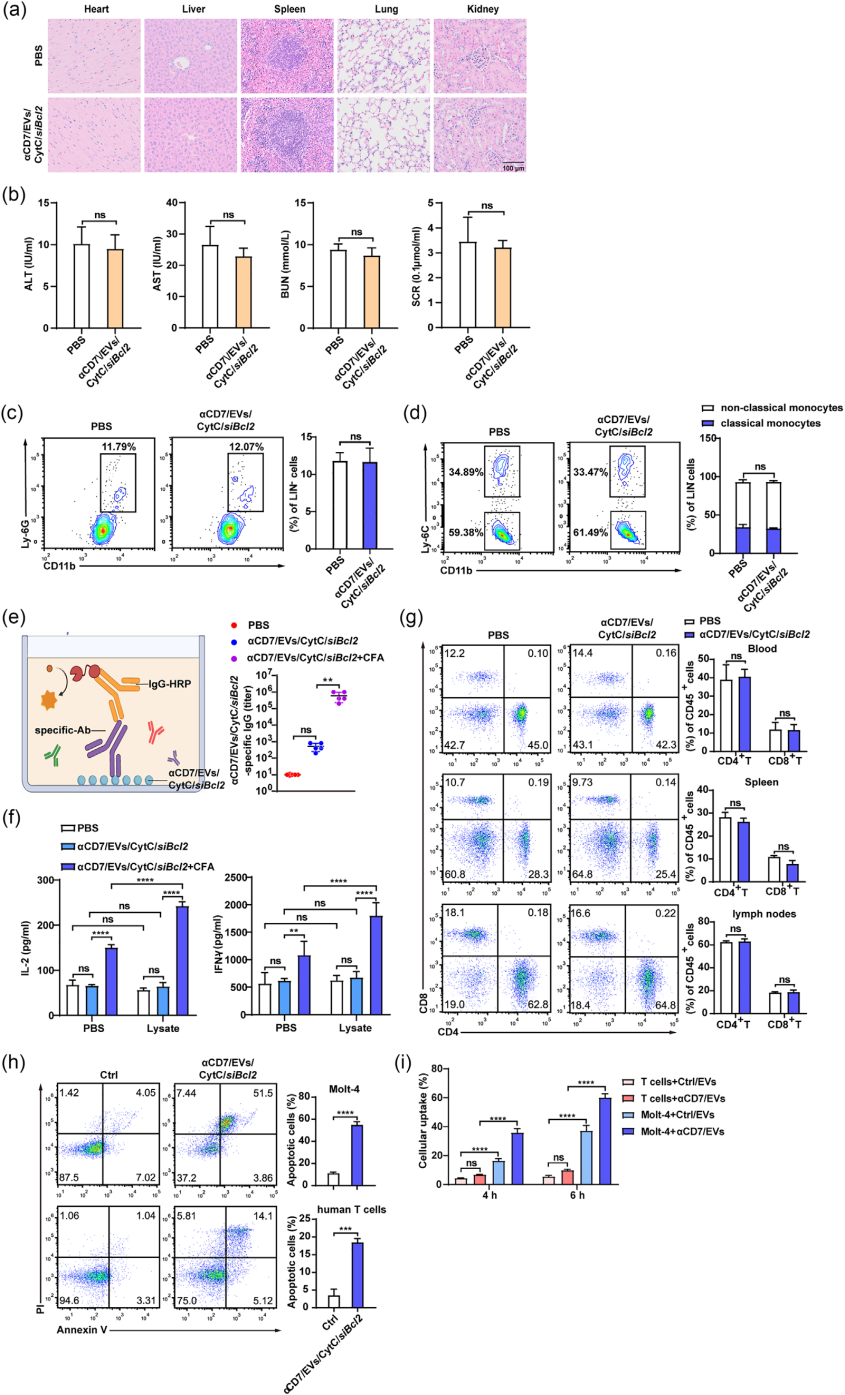
αCD7/EVs/CytC/*siBcl2* have a high biological safety and low immunogenicity. (a)–(d) BALB/c mice were intravenously injected with 100 µg (≈ 1.79 × 10^11^ particles) of αCD7/EVs/CytC/*siBcl2* every three days for a total of 5 times (*n* = 5). (a) Representative H&E staining images of the main organs of the mice. Scale bars, 100 µm. (b) Serum ALT, AST, BUN and SCR levels in the mice. (c) FACS analysis (left) and quantification (right) of neutrophils in the peripheral blood of the mice (LIN^−^ refers to CD3^−^ and B220^−^). (d) FACS analysis (left) and quantification (right) of Ly6C^hi^ classical monocytes and Ly6C^lo^ nonclassical monocytes in the peripheral blood of the mice (LIN^−^ refers to CD3^−^, B220^−^ and Ly6G^−^). (e) and (f) BALB/c mice were intravenously injected with 100 µg (≈ 1.79 × 10^11^ particles) of αCD7/EVs/CytC/*siBcl2* or subcutaneously injected with a mixture of αCD7/EVs/CytC*/siBcl2* with CFA. (e) Schematic diagram of the ELISA (left) and analysis (right) of the levels of αCD7/EV/CytC/*siBcl2*‐specific antibodies. (f) Splenocytes from the mice were isolated and restimulated with 5 µg of αCD7/EV/CytC/*siBcl2* lysates for 24 h. The levels of IL‐2 and IFN‐γ in the supernatants were detected by ELISA. (g) BALB/c mice were intravenously injected with 100 µg (≈ 1.79 × 10^11^ particles) of αCD7/EVs/CytC/*siBcl2* every three days for a total of 5 times (*n* = 5). FACS analysis (left) and quantification (right) of T cells in the spleen, lymph nodes and peripheral blood of the mice. (h) FACS analysis (left) and quantification (right) of apoptotic cells (%) in Molt‐4 and human T cells treated with 10 µg (≈ 1.79 × 10^10^ particles) of αCD7/EVs/CytC/*siBcl2*. (i) FACS analysis (left) and quantification (right) of the uptake of 1 µg (≈ 1.85 × 10^9^ particles) of PKH26‐labelled EVs by Molt‐4 and human T cells. The data are representative of three independent experiments. The error bars represent ± SDs (*p < 0.05, **p < 0.01, ***p < 0.001; unpaired Student's *t* test in [b]–[h]; one‐way ANOVA followed by multiple comparison tests in [e] and [i]; two‐way ANOVA followed by multiple comparison tests in [d] and [f]). CytC, cytochrome C; EVs, extracellular vesicles; FACS, fluorescence‐activated cell sorting.

To preliminarily explore the possibility of clinical translation, we evaluated the biological safety of αCD7/EVs/CytC/*siBcl2*. We found that repeated intravenous administration of αCD7/EVs/CytC/*siBcl2* did not result in any obvious histopathological damage to major organs of the mice, including the heart, liver, spleen, lungs and kidneys (Figure 6a). The therapy also had no adverse effects on liver or kidney function, as evidenced by the levels of alanine transaminase (ALT), aspartate aminotransferase (AST), urea nitrogen (BUN) and serum creatinine (SCR) in the serum (Figure 6b). Although αCD7/EVs/CytC/*siBcl2* induced intrinsic apoptosis, they had minimal effects on the levels of serum proinflammatory cytokines, including IL‐6 and TNF‐α (Figure S6a). These findings support the notion that αCD7/EVs/CytC/*siBcl2* exhibited minimal systemic toxicity and did not elicit acute inflammatory responses.

Furthermore, we measured the proportion of neutrophils and monocytes in the blood to analyze early immune responses and observed no significant changes following αCD7/EV/CytC/*siBcl2* treatment (Figure 6c,d). We subsequently evaluated the B‐cell‐mediated immune response and found no significant effect on the total levels of serum IgM and IgG (Figure S6b). Additionally, the ELISA we developed was used to detect αCD7/EV/CytC/*siBcl2*‐specific antibodies (Figure 6e). As a positive control, we found that subcutaneous injection of αCD7/EVs/CytC*/siBcl2* with complete Freund's adjuvant (CFA) notably induced αCD7/EV/CytC/*siBcl2*‐specific antibodies. However, intravenous injection of αCD7/EVs/CytC/*siBcl2* alone hardly induced a significant increase in the antibody level (Figure 6e). To evaluate the αCD7/EV/CytC/*siBcl2*‐specific T‐cell response, splenocytes from the above‐treated mice were restimulated with αCD7/EV/CytC/*siBcl2* lysates. Lysates were used to mitigate any potential proapoptotic effects associated with αCD7/EVs/CytC/*siBcl2*. Consistent with B‐cell responses, positive control splenocytes promoted IL‐2 and IFN‐γ production, while those from the experimental group did not (Figure 6f). These results indicate that repeated intravenous injections of αCD7/EVs/CytC/*siBcl2* minimally activated adaptive immune responses.

To test whether αCD7/EVs/CytC/*siBcl2* impair healthy T cells, we examined the proportions of T cells in the spleen, lymph nodes and peripheral blood of the mice treated as outlined above and found that their proportions were unchanged (Figure 6g). Furthermore, compared with Molt‐4 cells, human T cells isolated from peripheral blood mononuclear cells (PBMCs) and incubated with αCD7/EVs/CytC/*siBcl2* presented significantly lower apoptosis rates (Figure 6h). We hypothesized that this difference might be attributed to their EV uptake capacity. Our data indicated that human T cells showed a much lower uptake capacity for Ctrl/EVs and αCD7/EVs than Molt‐4 cells did. Although human T cells exhibited a slight increase in uptake of αCD7/EVs compared to that of the control group, the difference was not statistically significant (Figure 6i and Figure S6c). Collectively, these findings suggest that αCD7/EV/CytC/*siBcl2* therapy is safe, well tolerated by the immune system and healthy T cells, and has the potential for clinical application.

## 3 DISCUSSION

**FIGURE 7:**
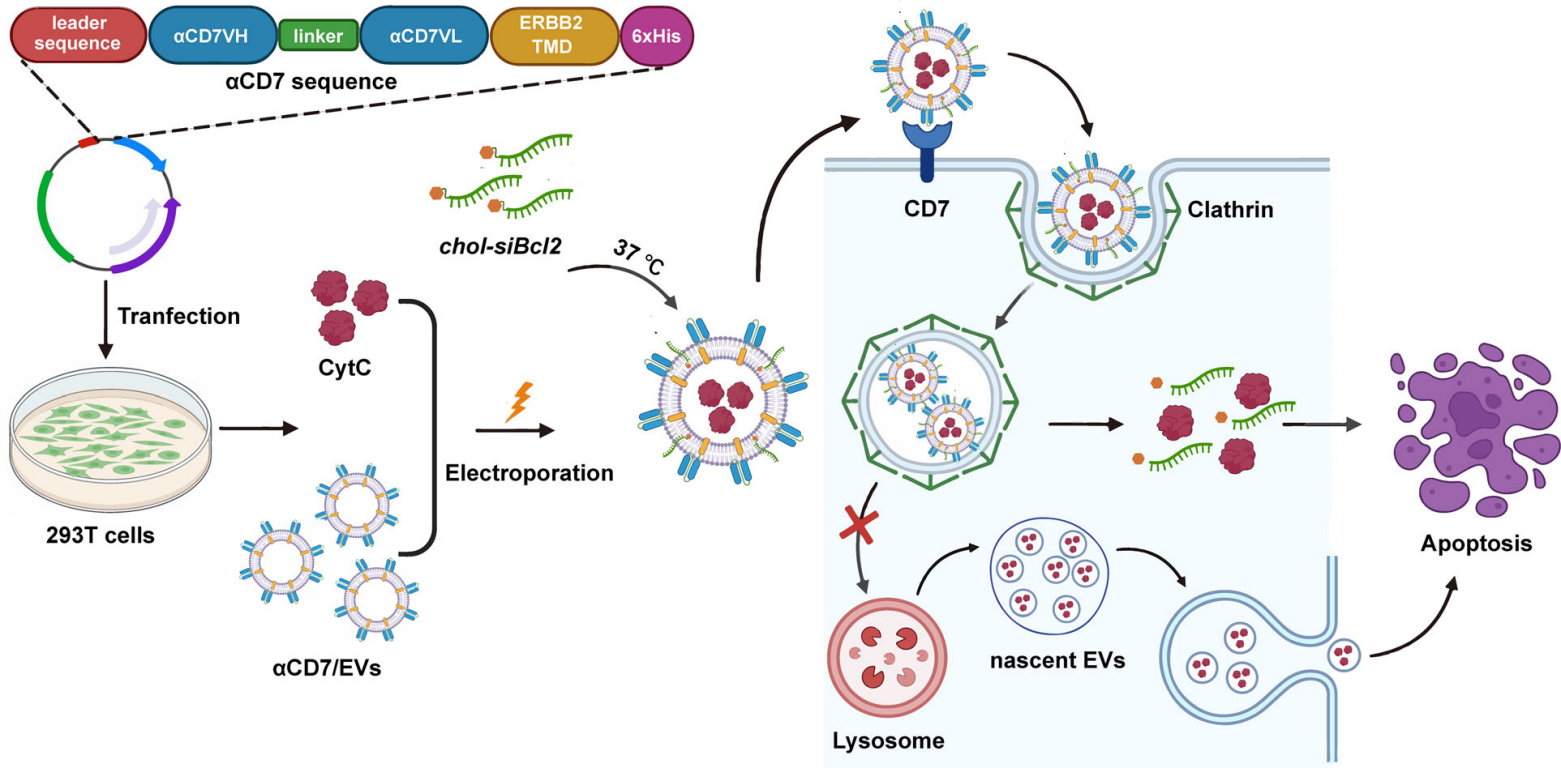
Schematic diagram of the use of αCD7/EVs/CytC/*siBcl2* for treating T‐cell malignancies.

Owing to the lack of biomarkers to distinguish malignant T cells from healthy T cells, CAR‐T‐cell therapy may lead to robust fratricide (Huang et al., 2023). CD7‐targeting CAR‐T cells are no exception. Thus, various techniques have been designed to produce CAR^CD7‐^ T cells, including CRISPR‐Cas9‐based genetic depletion of *CD7* (Cooper et al., 2018; Georgiadis et al., 2021) or inhibition of CD7 surface expression by IntraBlock technology (Huang et al., 2023; Pan et al., 2021; Png et al., 2017). Recent studies have focused on preselecting natural CD7‐negative T cells to manufacture CD7‐specific CAR‐T cells (Freiwan et al., 2022; Lu et al., 2022; Themeli, 2022). However, there is no doubt that these CD7 disruption administrations complicate the productive process and increase the cost and manufacturing resources. In addition, CD7 deficiency has been reported to reduce IL‐2 production in CAR‐T cells, thereby limiting their function due to low IL‐2 signaling (Hu et al., 2022). Instead of CD7‐targeting CAR‐T cells, we constructed αCD7/EVs with high levels of membrane‐associated αCD7 in this study and demonstrated that αCD7/EVs specifically targeted CD7^+^ Molt‐4 cells in vitro and in vivo (Figure 7). Unlike CAR‐T cells, which cannot distinguish between malignant and healthy T cells, human T cells from PBMCs treated with αCD7/EVs/CytC/*siBcl2* did not undergo apoptosis, which was likely related to the limited ability of T cells to take up 293T‐derived EVs. In addition, the modification of EVs is much simpler and less costly than the complicated gene editing of CAR‐T cells. Therefore, engineered EVs have robust potential for treating T‐ALL.

The efficiency of the EV drug delivery system is influenced to a certain degree by the endocytosis mechanism of recipient cells and the subsequent intracellular transport of these EVs. Owing to the diversity of recipient cells and the heterogeneity of EVs, the processes of endocytosis and intracellular transport are distinct. In this study, we confirmed that αCD7 modification altered the uptake of EVs by Molt‐4 cells from macropinocytosis‐dependent endocytosis to clathrin‐dependent endocytosis. Following the trafficking of EVs within cells, αCD7/EVs exhibited decreased colocalization with lysosomes, indicating that the drugs encapsulated in αCD7/EVs likely evaded lysosomal degradation and were packaged into nascent EVs of recipient cells (Figure 7). In fact, more CytC was found in nascent EVs from αCD7/CytC/EV‐treated Molt‐4 cells, which amplified the cytotoxicity of αCD7/CytC/EVs and facilitated their treatment of T‐ALL.

Despite the effectiveness of high‐dose chemotherapy regimens in treating T‐ALL, more than 40% of adult patients who are receiving chemotherapy experience relapse, which is associated mainly with drug resistance (Tian et al., 2020). A portion of patients also suffer from neurotoxic side effects (Śliwa‐Tytko et al., 2022). Therefore, we selected CytC as a potential therapeutic agent. CytC has been reported to induce apoptosis in cancer cells through diverse delivery systems, including fusion protein‐based platforms and nanoparticle‐mediated systems (Pessoa, 2022). However, whether it can be delivered via EVs is unclear. In this work, we demonstrated that CytC could be encapsulated within αCD7/EVs by electroporation and successfully induced the apoptosis of Molt‐4 cells, indicating that EVs are novel delivery vehicles for CytC. The Bcl2 family of proteins plays a critical role in regulating MOMP, and the clinical inhibitor of Bcl2, venetoclax, has demonstrated marked effectiveness in treating chronic lymphocytic leukaemia (Lew & Seymour, 2022). Consequently, we assumed that loading *siBcl2* could further enhance the apoptosis of Molt‐4 cells in combination with exogenous CytC carried by EVs. This hypothesis was supported by both in vitro and in vivo experiments. Notably, αCD7/EVs/CytC/*siBcl2* also exhibited therapeutic efficacy against CR‐Molt‐4 cells, underscoring the superior advantages of CytC and *siBcl2* over conventional chemotherapy drugs in treating drug‐resistant tumours. However, we observed that EVs loaded with only *siBcl2* did not induce Molt‐4 cell apoptosis. Recent studies have shown that MOMP is heterogeneous and can be characterized as complete MOMP, incomplete MOMP and minority MOMP, but only complete MOMP can lead to cell death (Cao et al., 2022; Guilbaud & Galluzzi, 2023; Kalkavan & Green, 2018). Therefore, the failure of *siBcl2* loaded alone to induce apoptosis may be attributed to the induction of incomplete MOMP.

As a cell‐free therapy, αCD7/EVs/CytC/*siBcl2* exhibited a remarkable safety profile with minimal toxicity, as evidenced by the absence of any signs of organ inflammation or functional injury. In addition, when αCD7/EVs/CytC/*siBcl2* were intravenously injected into mice, neither B‐cell‐mediated nor T‐cell‐mediated immune responses were triggered, as evidenced by measurements of IgG, IgM, IL‐2 and IFN‐γ levels. Notably, the activation of B and T cells was only achieved when αCD7/EVs/CytC/*siBcl2* were administered with complete Freund's adjuvant. More importantly, αCD7/EVs/CytC/*siBcl2* caused minor damage to healthy human T cells. Overall, these results indicate that αCD7/EVs/CytC/*siBcl2* are potential reagents for T‐ALL treatment.

We obtained αCD7/EVs through genetic engineering. (1) Following electroporation and coincubation, αCD7/EVs/CytC/*siBcl2* efficiently targeted and induced apoptosis in Molt‐4 cells. Additionally, we observed that αCD7/EVs primarily entered Molt‐4 cells via clathrin‐mediated endocytosis. (2) This specific endocytic pathway reduced the colocalization of EVs with lysosomes, leading to improved CytC delivery efficiency and enhanced cytotoxicity in nascent EVs derived from Molt‐4 cells treated with EVs. These two pathways synergized to eradicate Molt‐4 cells.

## 4 MATERIALS AND METHODS

### 4.1 Reagents

The information on all the reagents used in this study is listed in Table S1.

### 4.2 Human samples

Human blood samples from healthy volunteers were obtained from the Second Affiliated Hospital, Zhejiang University School of Medicine, which was approved by the Ethics Committee. All healthy volunteers were informed of the use of their samples, and signed consent forms were obtained.

### 4.3 Mice and cell lines

Female BALB/c and NSG mice (6–8 weeks old) were purchased from SLAC Laboratory Animal Co., Ltd. (Shanghai, China). The mice were housed under specific pathogen‐free conditions. All mouse experiments were conducted following the Animal Research Ethics Committee of Zhejiang University guidelines and under an approved protocol of Zhejiang University.

293T cells and the Raji human Burkitt lymphoma cell line were purchased from the Chinese Academy of Sciences (Beijin, China). The A549 human non‐small cell lung cancer cell line and HeLa cervical cancer cell line were purchased from the American Type Culture Collection (ATCC; Manassas, VA, USA). The human Molt‐4 T‐ALL cell line was purchased from Procell Life Science & Technology (Wuhan, Hubei, China). αCD7/293T, Molt‐4‐Luci, CR‐Molt‐4 and over‐Raji cells were established in our laboratory by lentivirus infection. 293T, αCD7/293T, A549 and HeLa cells were cultured in DMEM supplemented with 10% (v/v) FBS (Yeason, Shanghai, China) and 1% (v/v) penicillin/streptomycin. Raji, Molt‐4, Molt‐4‐Luci CR‐Molt‐4, Raji and over‐Raji cells were cultured in 1640 medium purchased from Procell Life Science & Technology. All the cells were cultured in a cell incubator at 37°C with 5% CO_2_.

### 4.4 Human T‐cell isolation

Human T cells were isolated from the PBMCs of healthy donors with the Human Biotin Positive Selection Kit II (STEMCELL, Vancouver, BC, V6A 1B6, Canada) according to the manufacturer's instructions, some of which were stimulated with plate‐bound anti‐CD3 (5 µg/mL, Bio X cell) and anti‐CD28 (5 µg/mL, BioLegend, San Diego, CA, USA) antibodies.

### 4.5 Plasmid construction and lentiviral transfection

The V_H_, V_L_ fragment of αCD7 was referenced from a patent (US10106609B2) and linked by a flexible (GGGGS) × 3 linker. The cDNA sequences for αCD7 tagged with His were synthesized by GENERAL BIO (Chuzhou, Anhui, China) and inserted into the lentiviral overexpression plasmid pCDH‐CMV‐MCS‐EF1‐copGFP backbone.

To generate αCD7/293T cells, 293T cells were initially seeded in 6‐cm dishes and subsequently transfected with the pCDH‐αCD7 expression plasmid and viral packaging plasmids with the JetPEI DNA transfection reagent (Polyplus, Strasbourg, France) following the manufacturer's protocol. After a 72‐h incubation period, the lentiviral supernatant was collected. Subsequently, the 293T cells were infected with the lentivirus in the presence of 10 µg/mL polybrene cotransfection reagent (HANBIO, Shanghai, China), in conjunction with centrifugation at 1500 × *g* for 2 h at 32°C. After 48 h, the αCD7/293T cells were sorted by GFP fluorescence with a Beckman Moflo Astrios EQ (Beckman Coulter, Brea, CA, USA).

To generate Molt‐4‐Luci, CR‐Molt‐4 and over‐Raji, Molt‐4 and Raji cells were incubated with the Luci, ABCB1 or CD7 lentivirus and centrifuged at 1000 × *g* for 2 h at 32°C. After 48 h, the Luci^+^ Molt‐4 cells were selected with 2 µg/mL blasticidin S (HANBIO), and the ABCB1^+^ Molt‐4 and over‐Raji cells were selected with 1 µg/mL puromycin (BBI Co., Ltd., Shanghai, China).

### 4.6 Isolation of EVs

293T cells and αCD7/293T cells were cultured in DMEM with 10% FBS, and the FBS was centrifuged at 100,000 × *g* for 90 min to eliminate preexisting FBS‐derived EVs. For the isolation of nEVs/αCD7 and nEVs/Ctrl, Molt‐4 cells were incubated with 10 µg of αCD7/EVs/CytC or 20 µg of Ctrl/EVs/CytC, respectively, for 24 h, after which the culture supernatant was discarded. After two washes with PBS, the cells were resuspended in a new EV‐free medium and cultured for 24 h. The culture supernatant was harvested and centrifuged at 300 × *g* for 10 min, 2000 × *g* for 20 min and 10,000 × *g* for 30 min at 4°C to remove cell debris. The large volume of the supernatant was concentrated with a 300 kDa molecular weight cutoff concentrator, whereas the small volume was not concentrated. All the supernatants were passed through a 0.22 µm syringe filter (Millipore). Afterwards, the supernatant was collected in 35 mL ultracentrifuge tubes (Beckman Coulter) and ultracentrifuged at 100,000 × *g* for 90 min at 4°C with an SW32 rotor. The resultant precipitate at the bottom of each tube was resuspended in PBS, collected into one ultracentrifuge tube and ultracentrifuged again. Eventually, the precipitate enriched with EVs was resuspended in PBS. The protein concentration of the EVs was determined with a BCA protein assay kit (Thermo Fisher Scientific; Waltham, CA, USA). The obtained EVs were stored at ‐80°C for later use.

### 4.7 Nanoparticle tracking analysis

For the measurement of particle size distribution and concentration, EVs were diluted to the proper concentration with PBS and evaluated with a NanoSight NS300 system (Malvern PANalytical, Shanghai, China) configured with a 488 nm laser and a high‐sensitivity sCMOS camera; the data were finally analyzed with NTA 3.3 software.

### 4.8 Electron microscopy

For negative EV staining, EVs were diluted to the proper concentration with PBS, and 200 mesh carbon films were hydrophilized with a glow discharge instrument at 15 mA for 25 s. Then, the EV solution was pipetted onto 200 mesh carbon‐coated copper grids and kept at room temperature (RT) for 1 min. After the excess suspension was removed with filter paper, the EVs were negatively stained with 2% uranyl acetate at RT for 1 min, and the excess suspension was removed and air dried. The EVs were assessed with a Nova Nano 450 electron microscope (Thermo Fisher Scientific).

### 4.9 Drug encapsulation

For loading of EVs with CytC (Solarbio, Beijing, China), 100 µg of purified EVs and 100 µg of CytC were gently mixed in electroporation buffer and transferred into 0.4 cm electroporation cuvettes (Bio‐Rad, Hercules, CA, USA). After electroporation by a BTX electroporator (Harvard Biosciences, Cambridge, MA, USA) at 200 V, 200 Ω and 500 µF in exponential decay wave mode, the mixture was incubated at 37°C for 1 h to ensure that the plasma membrane of the EVs had fully recovered. Free CytC was eliminated by ultracentrifugation at 120,000 × *g* for 20 min twice with an MLA 150 rotor. Density gradient ultracentrifugation was performed as a control to verify that free CytC was washed out. WB revealed that the CytC content in EVs was similar after ultracentrifugation (two rounds) and density gradient ultracentrifugation. The CytC loading efficiency was assessed by WB and calculated from the mass‒gray value standard curve.

For loading of EVs with *siBcl2* (GenePharma, Shanghai, China), 100 µg of EVs were incubated with 25 µg of single‐stranded *siBcl2* with 5′‐cholesterol and 3′‐FAM modification at 37°C for 1 h. Free *siBcl2* was eliminated by centrifugation at 120,000 × *g* for 20 min twice. The loading efficiency of *siBcl2* was assessed by measuring the FAM fluorescence signals with a SpectraMax iD5 plate reader (Molecular Devices, Silicon Valley, CA, USA) with excitation/emission at 485/525 nm and was calculated from a mass‐FAM fluorescence intensity standard curve.

### 4.10 Proteinase K digestion

To verify whether CytC was loaded into EVs, free CytC and αCD7/EVs/CytC were incubated with 10 µg/mL proteinase K at 4°C for 20 min. The digestion was subsequently terminated with PMSF, and 5 × SDS was added to prepare the WB samples.

### 4.11 FBS digestion

To verify whether *siBcl2* was loaded into EVs, free *siBcl2* and αCD7/EVs/*siBcl2* were incubated with equal volumes of FBS at 37°C for 4 h. The RNase present in FBS can degrade RNA, and the degradation of *siBcl2* was assessed by gel electrophoresis.

### 4.12 Flow cytometric analysis

For the staining of EVs, Ctrl/EVs and αCD7/EVs were combined with 4‐µm‐diameter aldehyde‐sulfate latex beads (Thermo Fisher Scientific) for 30 min at RT in PBS. Then, the mixture was blocked with 50 µL of EV‐free FBS for 30 min. After two washes with PBS at 3500 × *g* for 5 min, the bead‐coated EVs were stained with EV marker antibodies that were fluorescently labelled (BioLegend, San Diego, CA, USA) for 30 min at RT in the dark.

For analysis of the expression of αCD7 on the EV surface, 20 µg of αCD7/EVs was incubated with 1 µg of purified CD7‐His protein (ACROBiosystems, Beijing, China) at 37°C for 4 h on a 360° shaker. After two washes with PBS, CD7‐His‐loaded αCD7/EVs were subjected to the above surface staining steps and stained with a His fluorescent antibody (BioLegend).

For surface staining of the cells, single‐cell suspensions were washed with PBS twice and incubated with fluorescence‐labelled antibodies for 20 min at 4°C in the dark.

Cell apoptosis was analyzed with an apoptosis detection kit (MultiSciences, Hangzhou, Zhejiang, China). Briefly, the cells were resuspended in 1 binding buffer and stained with annexin V and PI antibodies for 15 min at 37°C.

After two washes with PBS, the cell‐ or bead‐coated EVs were analyzed by flow cytometry. All FACS data were acquired through a Cytoflex flow cytometer (Beckman Coulter) and analyzed with FlowJo software (Tree Star, Ashland, OR, USA).

### 4.13 Western blotting

Total protein was extracted from the cells with RIPA buffer (Thermo Fisher Scientific). EV proteins were lysed in 1× SDS. All protein samples were boiled for 10 min at 100°C. The samples were subsequently separated by SDS‒PAGE, transferred to polyvinylidene fluoride (PVDF) membranes (Millipore), and incubated with primary antibodies and HRP‐conjugated secondary antibodies [goat anti‐mouse IgG (H + L), goat anti‐rabbit IgG (H + L), MultiSciences]. Enhanced chemiluminescence reagents (4A Biotech, Beijing, China) and a Tanon 4500 Gel Imaging System (Tanon‐bio, Shanghai, China) were utilized to detect the bands.

### 4.14 EV labelling

For PKH26 labelling, EVs were resuspended in 250 µL of diluent C, and 1 µL of PKH26 ethanolic dye solution was added to another 250 µL of diluent C. Then, the two suspensions were mixed and incubated at RT for 5 min in the dark. Then, 500 µL of EV‐free FBS was added to terminate the staining. Then, the EVs were ultracentrifuged at 120,000 × *g* to remove the serum and washed twice with 1 mL of PBS. We collected the supernatants from each of the three ultracentrifugation steps and measured the fluorescence intensity of PKH26. Additionally, density gradient ultracentrifugation was performed as a control to verify that the free PKH26 was washed out. For VivoTrack 680 labelling, EVs were resuspended in 1 mL of PBS and mixed with 42 µM VivoTrack 680 at RT for 20 min in the dark.

### 4.15 Density gradient ultracentrifugation

The EVs/CytC‐ or PKH26‐labelled EVs were suspended in cold PBS and mixed with cold iodixanol to obtain a 36% iodixanol solution, which was added to the bottom of an ultracentrifuge tube. The 30%, 24%, 18% and 12% iodixanol solutions were prepared with cold PBS and successively layered in the ultracentrifuge tube to form a complete gradient. After ultracentrifugation at 120,000 × *g* for 16 h with an SW41 rotor, nine 1 mL fractions were collected from the top of the gradient. Each fraction was transferred to a new ultracentrifugation tube and ultracentrifuged at 120,000 × *g* for 4 h. The precipitate of each fraction was collected for WB or fluorescence intensity analysis.

### 4.16 Detection of EV uptake in vitro and in vivo

To monitor EV uptake by cells, PKH26‐labelled EVs were incubated with Molt‐4, Raji, A549, HeLa or human T cells for different periods, and the PE fluorescence intensity was detected by a Cytoflex flow cytometer. For immunofluorescence staining, Molt‐4, Raji, and human T cells were collected into a 1 mL EP tube and washed twice with PBS to remove the culture medium. After being fixed with 4% paraformaldehyde for 20 min and washed with PBS at 4000 × g for 5 min, the cells were stained with phalloidin iFluor™ 488 for 30 min. Finally, the cells were resuspended in an appropriate amount of DAPI (Vector Laboratories, Newark, CA, USA), after which 2 µL of the cell suspension was placed on a slide and covered with a coverslip for 2 h at RT or overnight at 4°C. The images of the cells were obtained with an Olympus IX83‐FV3000 confocal microscope (Olympus, Tokyo, Japan) and analyzed with ImageJ software.

To track EVs in vivo, 100 µg (≈ 1.79 × 10^11^ particles) of VivoTrack 680‐labelled EVs were intravenously injected into the mice. After 24 h, the mice were sacrificed, and the brain, heart, lungs, liver, spleen, kidneys, gut and tumour tissue were collected. Images were taken with an IVIS (PerkinElmer, Waltham, MA, USA).

To detect EV uptake in tumours, a fraction of tumour tissues was embedded in Tissue‐Tek O.C.T. compound (SAKURA, Torrance, CA, USA), followed by cryosection generation on a histological cryotome (Thermo CryoStar NX50). Then, the tissue sections were fixed in 4% paraformaldehyde for 20 min and stained with DAPI for 2 h at RT to stain the nuclei. The images of the sections were observed with an Olympus IX83‐FV3000 microscope.

### 4.17 Nanoflow

PKH26‐labelled or unlabelled EVs were suspended in PBS and diluted to a proper concentration (approximately 10^8^ particles/mL) in 100 µL of PBS. Before sample loading, quality control of the Flow NanoAnalyzer (NanoFCM, Xiamen, Fujian, China) was performed with scatterspheres (40–150 nm) and fluorospheres (Thermo Fisher). The nanoflow data were acquired and analyzed through the Flow NanoAnalyzer.

### 4.18 Cell viability assay

A total of 2 × 10^5^ Molt‐4 cells/well were seeded in 96‐well plates and cultured for 24 h. Then, 10 µg of CytC, 1.79 × 10^10^ particles of Ctrl/EVs, αCD7/EVs, Ctrl/EVs/CytC, and αCD7/EVs/CytC were added, and the mixture was incubated with the cells for 48 h. Viability was assayed with a Cell Counting Kit‐8 assay (TransGen, Beijing, China).

### 4.19 Immunohistochemistry staining

Molt‐4 tumour tissue sections were routinely deparaffinized and rehydrated, and antigen retrieval was performed using 10 mM sodium citrate buffer (pH 6.0). After being blocked with 5% BSA (BBI), the slides were incubated with antibodies against apoptosis‐related proteins (cleaved caspase 3 and 8, CST; cleaved caspase 9, Abbkine, Wuhan, Hubei, China) at 4°C overnight and the corresponding HRP‐conjugated secondary antibodies at RT for 30 min. Images were randomly acquired with an Olympus VS200 digital slice scanner (Olympus) and analyzed with ImageJ software (NIH, Bethesda, MD, USA).

### 4.20 Endocytosis of EVs analysis

To assess the endocytosis of EVs, 2 × 10^5^ Molt‐4 cells were pretreated in the presence or absence of 20 µM of the macropinocytosis inhibitor LY294002 (MedChemExpress, Monmouth Junction, NJ, USA), 80 mM of the caveolae‐mediated endocytosis inhibitor indomethacin (MedChemExpress) or 20 µM of the clathrin‐mediated endocytosis inhibitor chlorpromazine (MedChemExpress) for 1 h and incubated with 1 µg of PKH26‐labelled EVs for 4 h. The uptake of EVs was detected with a Cytoflex flow cytometer.

To detect the intracellular transport of endocytosed EVs, 3 × 10^6^ Molt‐4 cells were incubated with 15–20 µg of PKH26‐labelled EVs for 4 h. After two washes with PBS, the cells were fixed in 4% paraformaldehyde for 20 min at RT and washed with PBS. After being blocked with 5% BSA, the cells were incubated with antibodies against CD63, Lamp1 and clathrin at 4°C overnight. After three washes in PBS, the cells were incubated with DyLight 488‐conjugated secondary antibodies (HUABIO, Hangzhou, Zhejiang, China) at 4°C for 1 h. Finally, the cells were resuspended in DAPI for 2 h at RT to stain the nuclei. Images of the cells were obtained with an Olympus IX83‐FV3000 microscope. We analyzed the distributions of PKH26 and intensity at 488 nm for colocalization analysis across the ROI lines indicated in each merged image via the Plot Profile tool in ImageJ software. The Pearson's_Rr for each field was then calculated with the colocalization Finder tool in ImageJ software.

### 4.21 CytC‐containing nascent EVs from Molt‐4 cells in transwell chambers

To visualize CytC‐containing nascent EVs moving from treated Molt‐4 cells to adjacent Molt‐4 cells, CytC was labelled with an Alexa Fluor 680 protein labelling kit (Thermo Fisher) and then loaded into αCD7/EVs by electroporation. A total of 5 × 10^5^ Molt‐4 cells were pretreated with or without 5 µM of SMasei for 2 h, incubated with 20 µg (approximately 3.58 × 10^10^ particles) of αCD7/EVs/CytC^680^ for 12 h, washed with PBS to eliminate exogenous EVs and then placed in the upper chamber. Blank Molt‐4 cells (2.5 × 10^5^) were placed in the lower chamber and incubated in the upper chambers for 24 h. Fluorescence in the nascent EVs and blank Molt‐4 cells was detected by flow cytometric analysis and an Olympus IX83‐FV3000 microscope.

### 4.22 Inhibition of EV production

To reduce EV production, Molt‐4 cells were treated in the presence or absence of 5 µM SMasei (Santa Cruz, Dallas, Texas, USA) for 24 h. NTA and flow cytometric analysis were used to assess the production of EVs. To inhibit EV production from Molt‐4 tumours, 2.5 µg/g SMasei (Santa Cruz) was intratumourally injected 24 h before treatment with drug‐loaded EVs.

For analysis of the production of EVs from Molt‐4 tumours, 1.5 g Molt‐4 tumour tissues were dissected and digested with 1 mg/mL collagenase type IV (Worthington Biochemical) and 0.1 ng/mL DNase I (Sigma–Aldrich) in 10% EV‐free FBS RPMI‐1640 medium at 37°C for 2 h on a 360° shaker. The EVs in the digestive supernatant were harvested by ultracentrifugation and detected with BCA and NTA.

### 4.23 TUNEL analysis

The TUNEL assay was conducted with a TUNEL detection kit (TUNEL, Beyotime Biotechnology, Shanghai, China) according to the manufacturer's instructions. Briefly, tumour slices were fixed in 4% paraformaldehyde at RT for 20 min. After two washes with PBS, the samples were treated with 0.3% Triton X‐100 for 15 min. Subsequently, 50 µL of the TUNEL reaction mixture (5 µL of TdT enzyme solution and 45 µL of labelling solution) was added, and the samples were incubated for 1 h at 37°C in the dark. The sections were stained with DAPI and visualized with an Olympus IX83‐FV3000 microscope.

### 4.24 Xenograft mouse models

A total of 1.3 × 10^7^ Molt‐4, CR‐Molt‐4 or Raji cells were subcutaneously injected into the right flank of female NSG mice. After approximately two weeks, the mice with tumours were randomly divided into several groups and intravenously injected with PBS, 100 µg of CytC, 1.79 × 10^11^ particles of Ctrl/EVs, αCD7/EVs, Ctrl/EVs/CytC, αCD7/EVs/CytC, Ctrl/EVs/CytC/*siBcl2*, or αCD7/EVs/CytC*/siBcl2*, or 2.5 µg/g Dox every three days. The tumour volume was measured with a Vernier caliper throughout the treatment cycle.

To develop a T‐ALL mouse model, 5 × 10^6^ Molt‐4‐Luci cells or Molt‐4‐Luci CR‐Molt‐4 mixed cells were intravenously injected into NSG mice. Approximately one week later, the mice with tumours were randomly divided into three groups and intravenously injected with PBS, 1.79 × 10^11^ particles of Ctrl/EVs/CytC/*siBcl2*, αCD7/EVs/CytC/*siBcl2*, 2.5 µg/g Dox or 2.5 µg/g Dox + 1.79 × 10^11^ particles of αCD7/EVs/CytC/*siBcl2* every three days. Tumour growth was monitored by IVIS after intraperitoneal injection of 150 mg/kg luciferin (Promega, Beijing, China) and analyzed with Living Image version 4.4 software (Caliper Life Sciences).

### 4.25 Serum biochemistry

Mouse blood was allowed to settle for 2 h at RT and then centrifuged at 1500 × *g* for 15 min to collect clear serum. The activities of serum ALT, AST, urea nitrogen and CR were measured with ALT, AST, BUN and SCR Reagent Kits (Jiancheng, Nanjing, Jiangsu, China), respectively. The levels of IgG, IgM, TNFα and IL‐6 in the serum were measured by ELISA kits (Thermo Fisher Scientific) according to the manufacturer's instructions.

### 4.26 Detection of drug‐loaded EV‐specific antibodies

To evaluate the immunogenicity of αCD7/EVs/CytC/*siBcl2*, female BALB/c mice were randomly divided into three groups and intravenously injected with PBS or 1.79 × 10^11^ particles of αCD7/EVs/CytC/*siBcl2* or subcutaneously injected with 1.79 × 10^11^ particles of αCD7/EVs/CytC/*siBcl2* mixed with complete Freund's adjuvant once a week for a total of three times. To prepare complete Freund's adjuvant, 10 mL of incomplete Freund's adjuvant (Sigma–Aldrich) was mixed with 100 mg of nonviable *Mycobacterium tuberculosis* H37 Ra (BD, Franklin Lake, NJ, USA). After 2 weeks, the mice were sacrificed, and their serum was harvested. The levels of αCD7/EV‐specific antibodies were measured by ELISA. Briefly, 96‐well ELISA plates (Corning, NY, New York, USA) were coated with 4 µg/mL αCD7/EVs/CytC/*siBcl2* in coating buffer (Thermo Fisher Scientific) and incubated overnight at 4°C. After being blocked with assay diluent (Thermo Fisher Scientific), the EVs were fixed and permeabilized with IC Fixation (Thermo Fisher Scientific) and Permeabilization Buffer (Thermo Fisher Scientific). Then, the serum samples were diluted with blocking buffer, added to individual wells and incubated overnight at 4°C. The plates were washed and incubated with goat anti‐mouse IgG (H+L) (MultiSciences) (1:5000) in blocking buffer for 2 h at RT. Then, the signal was developed with TMB, and the samples were blocked with 2 M H_2_SO_4_, after which the absorbance at 450 nm was measured with a SpectraMax iD5 microplate reader (Molecular Devices, San Francisco, CA, USA).

### 4.27 Statistical analysis

All the data are expressed as mean ± s.d. values. Statistical analyses were performed with GraphPad Prism 8.0 software. Unpaired Student's *t* test was used to analyze the significance of the average differences between two groups, and differences among multiple groups was analyzed by one‐way or two‐way ANOVA followed by Tukey's test. A difference was considered significant if the *P* value was < 0.05. All the error bars of the experimental data are presented as the SDs.

## AUTHOR CONTRIBUTIONS

Bei Zhang, Jianqiang Chen, Jiming Chen, Yingying Shen, Yinghu Chen, Shibo Wang and Chengyan Zhang performed various experiments. Yuzhou He, Huajun Feng, Jiaoli Wang and Zhijian Cai designed the project and supervised the study. Bei Zhang and Zhijian Cai analyzed the data and wrote the manuscript. Yingying Shen participated in the the human T‐cell isolation experiments and the establishment of xenograft mouse models. Yinghu Chen participated in the nanoparticle tracking analysis and EV‐specific antibody detection experiments. Shibo Wang participated in the nanoflow and proteinase K digestion experiments. Chengyan Zhang participated in the EV labelling experiments and cell viability assay. Jiming Chen participated in in vivo animal experiment, the immunohistochemistry staining and density gradient ultracentrifugation experiments. Yuzhou He participated in the project design and guidance. Huajun Feng participated in the project design and guidance. Jiaoli Wang participated in the project design and guidance. Zhijian Cai designed the project, supervised the study and revised the manuscript.

## CONFLICT OF INTEREST STATEMENT

The authors declare no competing interests.

## Supporting information



Supporting Information
