# Supplementary material for: CD7‐targeting pro‐apoptotic extracellular vesicles: A novel approach for T‐cell haematological malignancy therapy
Source: J Extracell Vesicles. 2024 Dec 16;13(12):e70025. doi: 10.1002/jev2.70025 (PMC11647336; doi:10.1002/jev2.70025)
Supplement: Supplementary file 1 — Supporting Information [file JEV2-13-e70025-s001.docx]

**
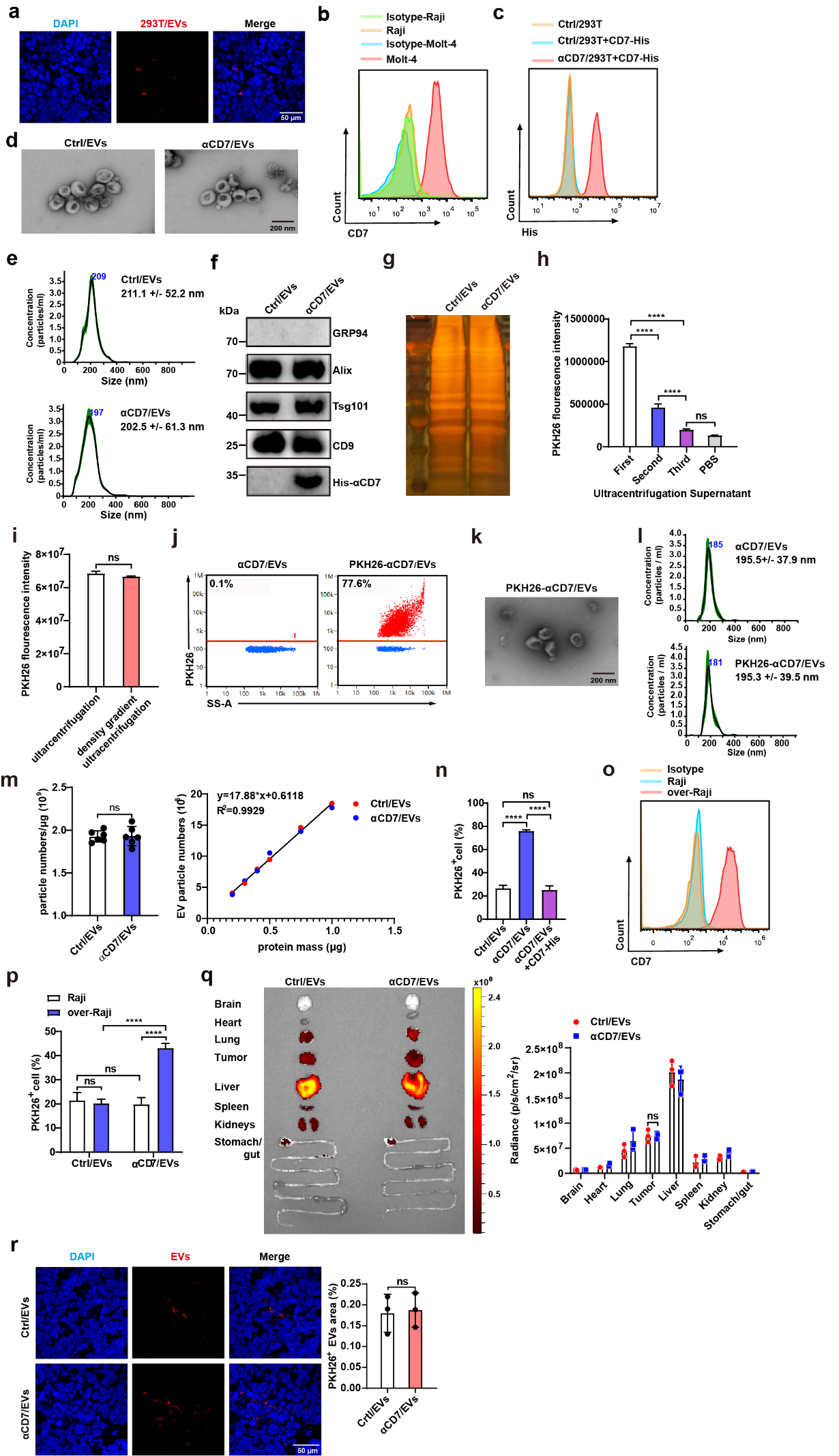
**

**Fig. S1** Anchorage of αCD7 on EVs promotes EVs uptake by CD7^+^ T-cell malignancy. **a**, Representative images of Molt-4 tumor sections from the NSG mice intravenously injected with 100 μg VivoTrack 680-labeled 293T/EVs for 24 h. Scale bars, 50 μm (n = 3). **b**, FCAS analysis of CD7 level on Raji and Molt-4 cells (n = 3). **c**, 1 μg CD7-His co-incubated with αCD7 stable expressing 293T cells (αCD7/293T) and 293T cells with mock transfection (Ctrl/293T). FCAS analysis of αCD7 levels on αCD7/293T cells and Ctrl/293T by detecting His (n = 3). **d**, EM detection of Ctrl/EVs and αCD7/EVs morphology. Scale bar, 200 nm. **e**, NTA analysis of Ctrl/EVs and αCD7/EVs particle distribution. **f**, WB analysis of the indicated EV markers and His-αCD7 in Ctrl/EVs and αCD7/EVs. **g**, Silver staining of equal amounts of Ctrl/EVs and αCD7/EVs. **h**, Fluorescence intensity of PKH26 from the supernatants from each of the three ultracentrifugation steps following EVs labeling with PKH26. PBS was used as negative control **i**, PKH26 fluorescence intensity of equal amounts of αCD7/EVs after ultracentrifugation or density gradient ultracentrifugation. **j**, Nanoflow analysis of PKH26^+^ αCD7/EVs. **k**, EM detection of PKH26-labeled αCD7/EVs morphology. Scale bar, 200 nm. **l**, NTA analysis of PKH26 unlabeled or labeled αCD7/EVs particle distributions. **m**, Particle numbers per unit mass of Ctrl/EVs and αCD7/EVs (left). Mass-particle number standard curve of Ctrl/EVs and αCD7/EVs (right). **n**, FCAS quantification of 1 μg particles PKH26-labeled EV (Ctrl/EVs, αCD7/EVs, αCD7/EVss + CD7-His) uptake by Molt-4 cells for 4h. **o**, FCAS analysis of CD7 level on Raji and over-Raji cells (n = 3). **p**, FCAS quantification of 1 μg (≈ 1.85 × 10^9^ ) PKH26-labeled EV (Ctrl/EVs, αCD7/EVs) uptake by Raji cells and over-Raji cells for 1 h. (**q**, **r**) Raji tumor-bearing NSG mice were intravenously injected with 100 μg VivoTrack 680-labeled Ctrl/EVs or αCD7/EVs for 24 h (n = 3). **q**, Representative IVIS images (left) and quantification (right) of EV uptake in the indicated organs and tumors. **r**, Representative confocal images (left) and quantification (right) of VivoTrack 680-labeled EVs in Raji tumor sections. Scale bars, 50 μm. Each dot indicates a randomly acquired image. Data are representative of three independent experiments. Error bars represent mean ± SD (ns, not significant, **p* < 0.05, ***p* < 0.01, *****p* < 0.0001, one-way ANOVA followed by Turkey’s test in **h**, **n**, two-way ANOVA followed by multiple comparison test in **p**, unpaired Student’s *t*-test in **i**, **m**, **r**).

**
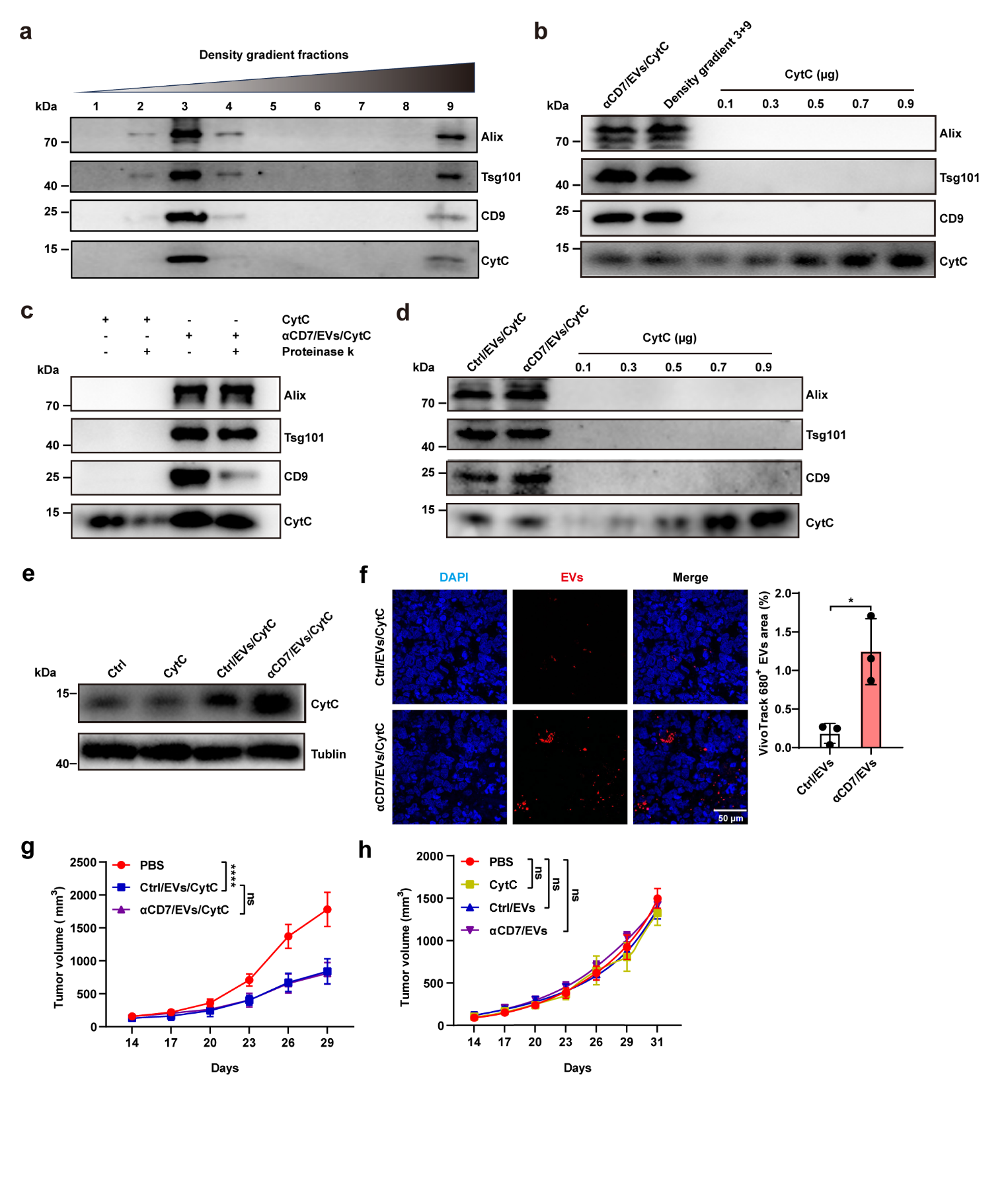
**

**Fig. S2** αCD7/EVs loaded with CytC effectively inhibit CD7^+^ T-cell malignancy. **a**, WB analysis of the indicated EV markers and CytC in each 1 ml iodixanol gradient fraction. **b**, WB quantification of CytC in 5 μg αCD7/EVs/CytC after ultracentrifugation twice or density gradient ultracentrifugation. **c**, WB analysis of the indicated EV markers and CytC after being treated with proteinase k. **d**, WB quantification of CytC in 5 μg Ctrl/EVs/CytC and αCD7/EVs/CytC after ultracentrifugation twice. **e**, WB analysis of CytC in Molt-4 cells treated with Ctrl/EVs/CytC or αCD7/EVs/CytC for 24 h. **f**, Representative images of Molt-4 tumor sections from the NSG mice intravenously injected with 100 μg (≈ 1.79 × 10^11^ particles) VivoTrack 680-labeled Ctrl/EVs/CytC or αCD7/EVs/CytC for 24 h. Scale bars, 50 μm (n = 3). **g**, NSG mice were subcutaneously injected with Raji cells and intravenously injected with PBS, 100 μg (≈ 1.79 × 10^11^ particles) Ctrl/EVs/CytC or αCD7/EVs/CytC every three days for 5 times (n = 5). Tumor progression was evaluated based on the tumor size. **h**, NSG mice were subcutaneously injected with Molt-4 cells and intravenously injected with PBS, 100 μg CytC, 100 μg (≈ 1.79 × 10^11^ particles) Ctrl/EVs or αCD7/EVs every three days for 5 times (n = 5). Tumor progression was evaluated based on the tumor size. Data are representative of three independent experiments. Error bars represent mean ± SD (ns, not significant, *p < 0.05, ****p < 0.0001, unpaired Student’s *t*-test in **f**, one-way ANOVA followed by Turkey’s test in **g**, **h**).

**
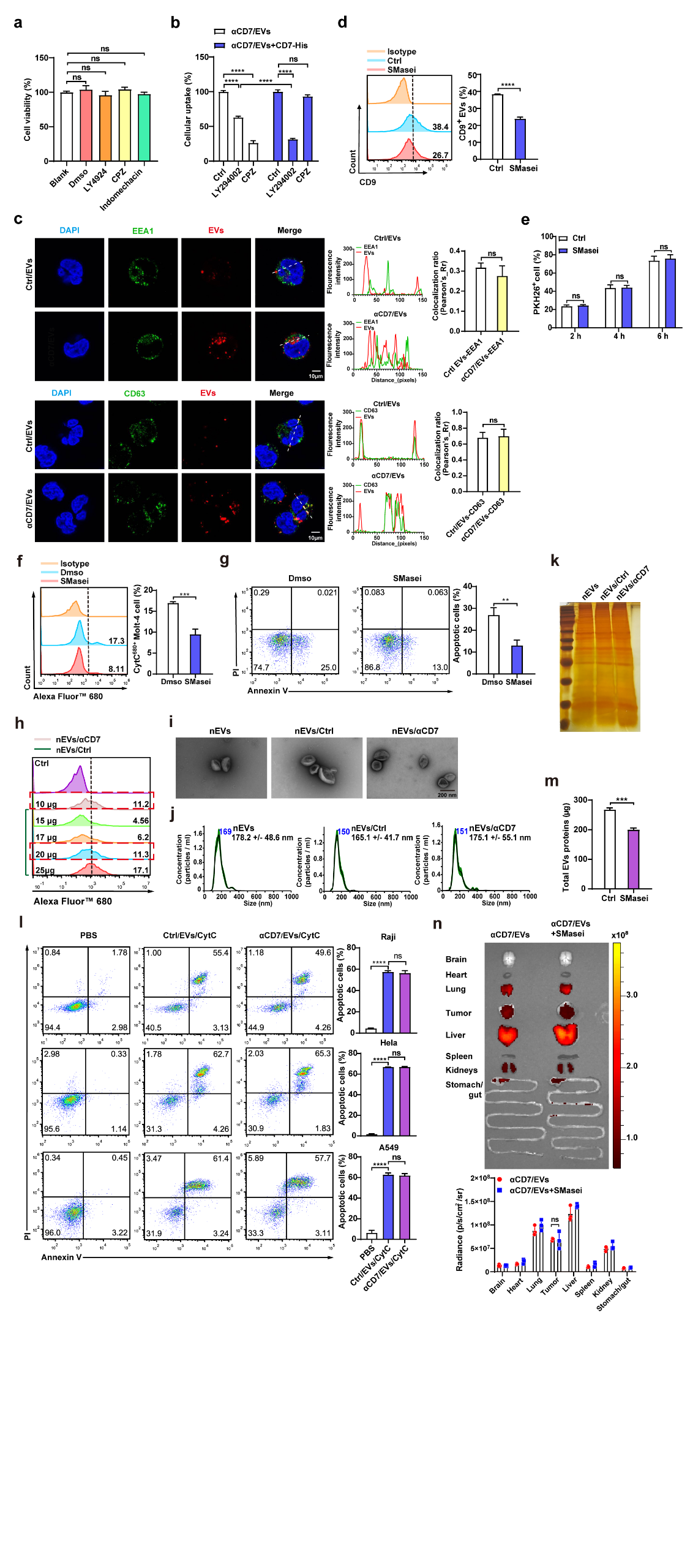
**

**Fig. S3** αCD7 modification alters EV endocytosis fashion and facilitates offspring EV cytotoxicity **a**, The viability of Molt-4 cells treated with the indicated endocytosis inhibitors for 24 h was analyzed by CCK8 assay (n = 3). **b**, 20 μg (≈ 3.58 × 10^10^ particles) αCD7/EVs were incubated with 1 μg CD7 Ag for 4 h at 37 ℃. FCAS analysis of the 4 h uptake of 1 μg PKH26-labeled αCD7/EVs by Molt-4 cells pre-treated with the indicated endocytosis inhibitors for 1 h (n = 3). **c**, Representative confocal images (left) of the colocalization of PKH26-labeled EVs with endosome-related proteins (EEA1 and CD63, green). Scale bars, 10 μm. Fluorescence profile analysis and colocalization ratio analysis (right) of PKH26-labeled EVs and endosome-related proteins (EEA1 and CD63, green) (n =3 ). **d**, Molt-4 cells were treated with 5 μM SMasei or without for 24 h. EVs in the supernatants were captured by anti-CD63-coated latex beads, and FCAS analyzed CD9-positive beads to indicate EV release (n = 3). **e**, FCAS analysis of the 4 h uptake of 1 μg (≈ 1.85 × 10^9^ particles) PKH26-labeled αCD7/EVs by Molt-4 cells pre-treated with 5 μM SMasei for 2 h, 4 h or 6 h (n = 3). **f**, FCAS analysis (left) and quantification (right) of CytC^680^ in lower Molt-4 cells (n = 3). **g**, FCAS analysis (left) and quantification (right) of the apoptotic cells (%) of lower Molt-4 cells (n = 3). **h**, FCAS analysis of 12 h uptake of the indicated Ctrl/EVs/CytC^680^ doses and 10 μg nEVs/αCD7^680^ by Molt-4 cells. **i**, EM detection of nEVs, nEVs/Ctrl and nEVs/αCD7 morphology. Scale bar, 200 nm. **j**, NTA analysis of nEVs, nEVs/Ctrl and nEVs/αCD7 particle distribution. **k**, Silver staining of equal amounts of nEVs, nEVs/Ctrl and nEVs/αCD7. **l**, FCAS analysis (left) and quantification (right) of the pro-apoptotic effect of 10 μg (≈ 1.79 × 10^10^ particles) Ctrl/EVs/CytC or αCD7/EVs/CytC on the indicated tumor cells (n = 3). **m**, BCA assay was used to determine the amount of proteins in EVs from 1.5 g Molt-4 tumors injected with or without 2.5 μg/g SMasei 5 times (n = 3). **n**, Representative IVIS images (up) and quantification (bottom) of VivoTrack 680-labeled αCD7/EVs uptake in the indicated organs and tumors pre-treated with SMasei (n = 3). Data are representative of three independent experiments. Error bars represent ± SD (ns, not significant, ****p* < 0.001, *****p* < 0.0001, unpaired Student’s *t*-test in **a**, **c**, **d**, **e**, **f**, **g**, **m**, **n**, two-way ANOVA followed by Turkey’s test in **b**, one-way ANOVA followed by Turkey’s test in **l**).

**
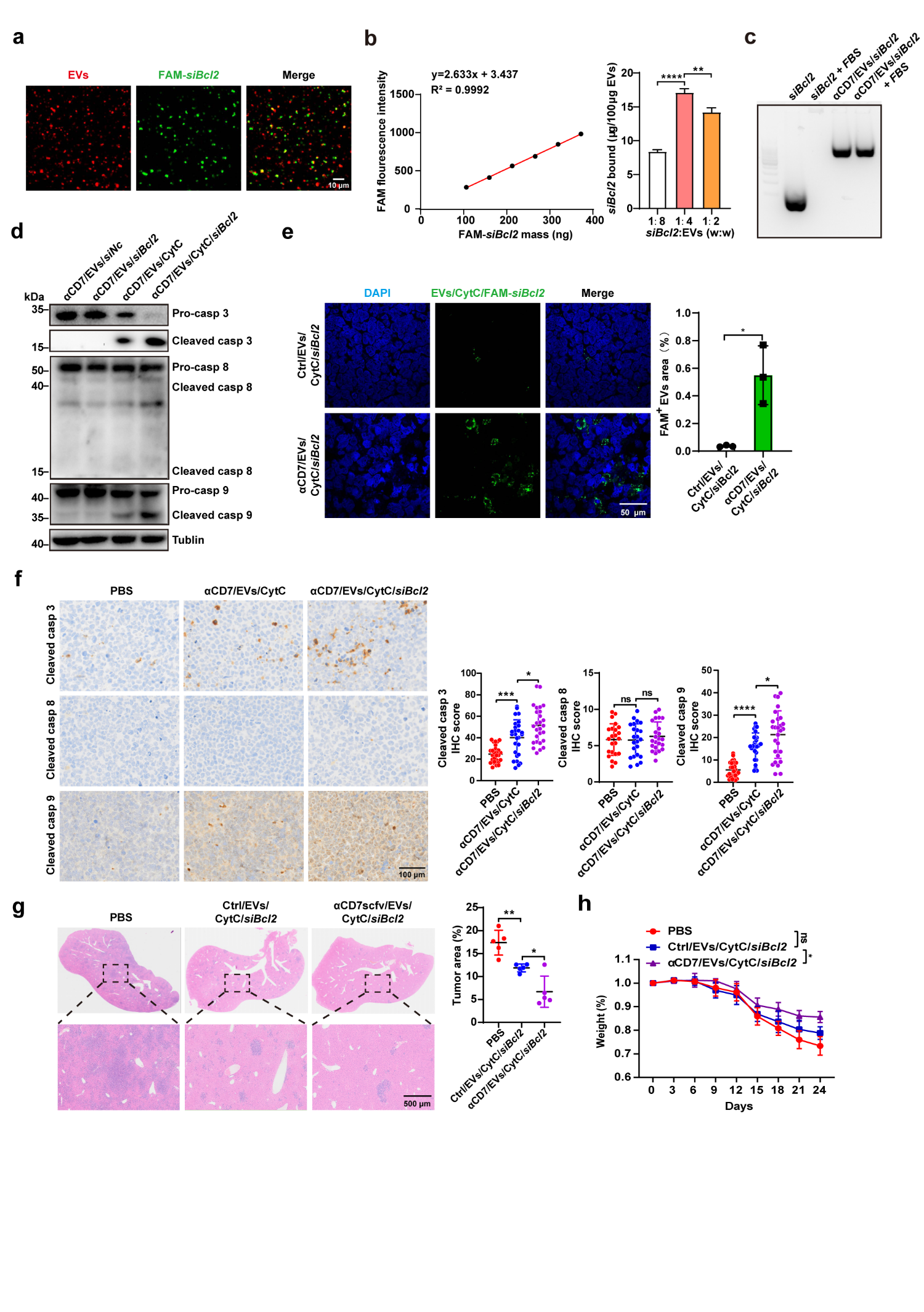
**

**Fig. S4** Bcl2 silencing enhances the therapeutic effects of αCD7/EVs/CytC on CD7^+^ T-cell malignancy. (**a**, **b**) The *siBcl2* was decorated with 5’-cholesterol and 3’-FAM. **a**, Representative confocal images of the colocalization of PKH26-labeled EVs with *siBcl2*. **b***, siBcl2* mass-fluorescence intensity standard curve (left) and *siBcl2* calculation in 100 μg αCD7/EVs/CytC (right) (n = 3). **c**,  Gel electrophoresis analysis of free *siBcl2* and αCD7/EVs/*siBcl2* degradation by FBS for 4 h. **d**, WB analysis of the protein markers of apoptosis in Molt-4 cells treated with αCD7/EVs/*siBcl2*, αCD7/EVs/CytC or αCD7/EVs/CytC/*siBcl2* for 48 h. **e**, Representative confocal images (left) and quantification (right) of FAM signals in Molt-4 tumor sections from the NSG mice intravenously injected with 100 μg (≈ 1.79 × 10^11^ particles) Ctrl/EVs/CytC/*siBcl2* or αCD7/EVs/CytC/*siBcl2* for 24 h. The *siBcl2* was decorated with 5’-cholesterol and 3’-FAM. Scale bars, 50 μm (n = 3). **f**, Representative IHC images (left) and quantification (right) of cleaved caspase 3, 8 and 9 proteins in Molt-4 tumor tissues from the tumor-bearing mice (n = 5). Scale bars, 100 μm. Each dot indicates a randomly acquired image (n = 27-30). (**g, h**) NSG mice with T-ALL were intravenously injected with 100 μg (≈ 1.79 × 10^11^ particles) Ctrl/EVs/CytC/*siBcl2*  or αCD7/EVs/CytC/*siBcl2* every 3 days for 5 times (n = 5). **g**, Representative H&E staining images (left) and quantification (right) of the infiltration of T-ALL in liver lobes (n = 5). **h**, Body weights of the T-ALL mice (n = 5). Data are representative of three independent experiments. Error bars represent ± SD (ns, not significant, **p* < 0.05, ***p* < 0.01, ****p* < 0.001, *****p* < 0.0001, one-way ANOVA followed by Turkey’s test in **b**, **f**, **g**, **h**, unpaired Student’s *t*-test in **e**).

**
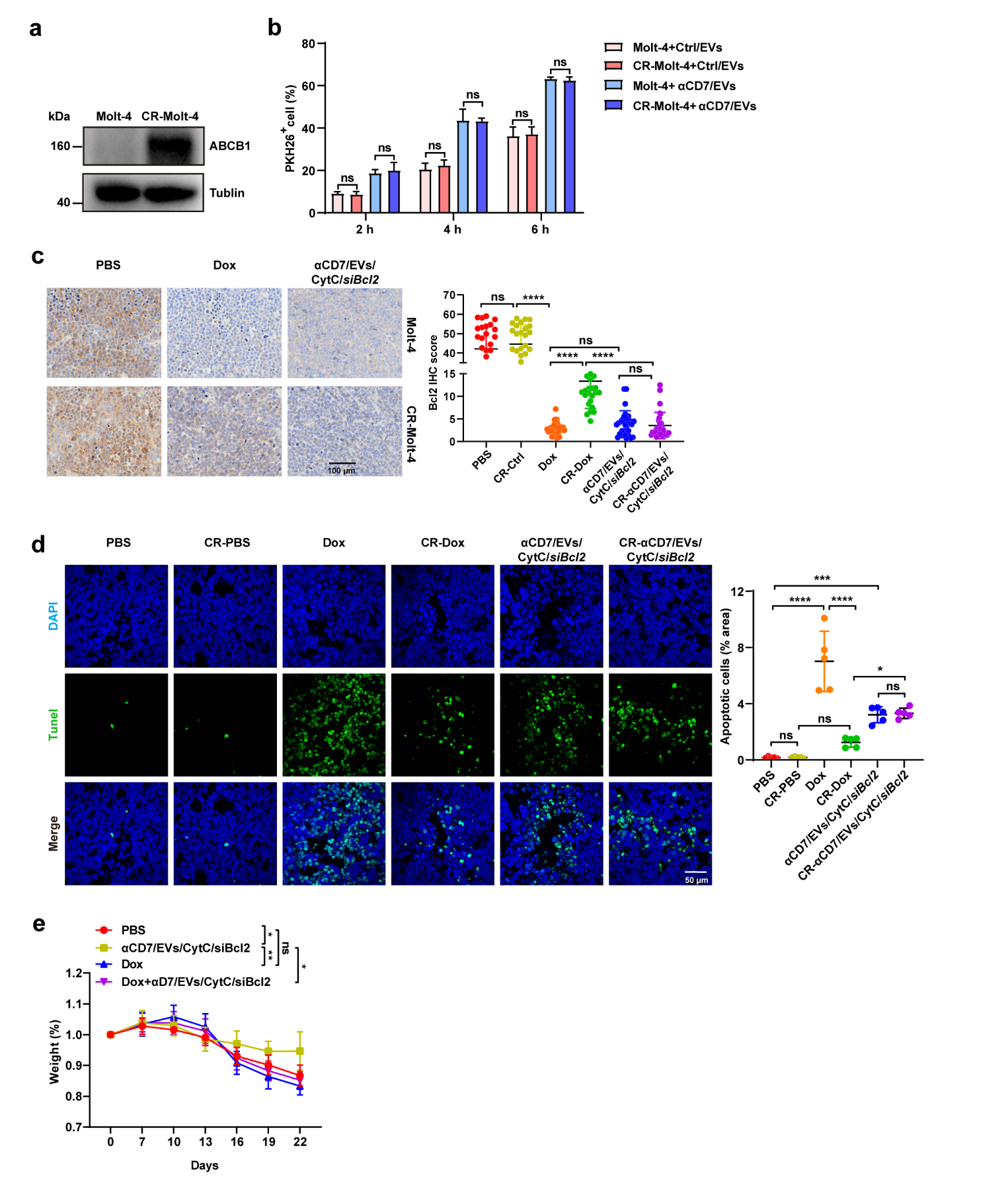
**

**Fig. S5** αCD7/EVs/CytC/*siBcl2* effectively inhibit chemotherapy-resistant CD7^+^ T-cell malignancy. **a**, WB analysis of ABCB1 protein levels in Molt-4 cells and CR-Molt-4 cells. **b**, FCAS analysis of 1 μg (≈ 1.85 × 10^9^ particles) PKH-26-labeled Ctrl/EVs or αCD7/EVs uptake by Molt-4 cells and CR-Molt-4 cells (n = 3). **c**, Representative IHC images (left) and quantification (right) of Bcl2 protein in tumor tissues from the mice (n = 5). Scale bars, 100 μm. Each dot indicates a randomly acquired image. **d**, Representative images (left) of TUNEL immunofluorescence staining of Molt-4 tumor tissues from the mice and quantification (right) of apoptotic cells per reported area Scale bars, 50 μm. Each dot indicates a randomly acquired image (n = 5). **e**, Body weights of the T-ALL mice (n = 5). Data are representative of three independent experiments. Error bars represent ± SD (ns, not significant, *****p* < 0.0001, unpaired Student’s *t*-test in **b**, one-way ANOVA followed by Turkey’s test in **c, d, e**).

**
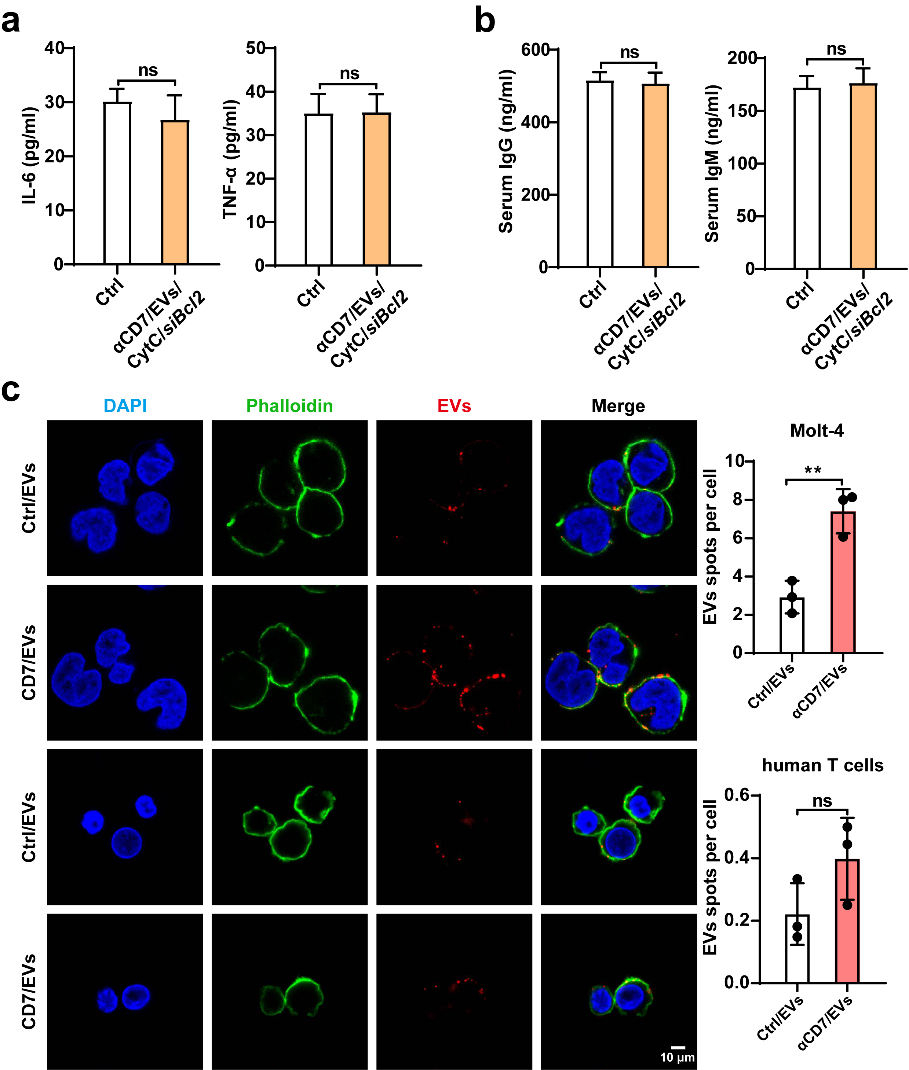
**

**Fig. S6** αCD7/EVs/CytC/*siBcl2* have high biological safety and low immunogenicity. (**a**, **b**) BALB/c mice were intravenously injected with 100 μg (≈ 1.79 × 10^11^ particles) αCD7/EVs/CytC/*Bcl2* every three days for a total of 5 times (n = 5). **a**, ELISA detection of IL-6 and TNF-α levels in the serum of these mice. **b**, ELISA detection of IgM and IgG levels in the serum of these mice. **c**, Representative confocal images (left) and quantification (right) of the PKH-26-labeled EV (red) uptake by Molt-4 and human T cells (green) for 4 h. Scale bars, 10 μm. Each dot indicates the number of EV spots per cell. Data are representative of three independent experiments. Error bars represent ± SD (ns, not significant, ***p* < 0.01, unpaired Student’s *t*-test in **a**-**c**).

**Supplementary Table 1**

| **REAGENT or RESOURCE** | **SOURCE** | **IDENTIFIER** |
| --- | --- | --- |
| **Antibodies** | | |
| anti-mouse CD9 APC | BioLegend | Cat: 124812 |
| anti-human CD9 APC | BioLegend | Cat: 312108 |
| anti-mouse CD45 PB | BioLegend | Cat: 103126 |
| anti-mouse CD4 PB | BioLegend | Cat: 100235 |
| anti-mouse CD4 APC | BioLegend | Cat: 100412 |
| anti-mouse CD8α PE | BioLegend | Cat: 135205 |
| anti-mouse CD8α APC | BioLegend | Cat: 119703 |
| anti-mouse B220 PE | BioLegend | Cat: 103208 |
| Ghost Dye™ Blue 516 | TONBO biosciences | Cat: 13-0867 |
| anti-human CD7 APC | BioLegend | Cat: 395605 |
| anti-His Tag APC | BioLegend | Cat: 362605 |
| Annexin V APC | MultiSciences | Cat: AP107-100 |
| PE PI | MultiSciences | Cat: AP107-100 |
| Rabbit anti-CD9 | Abclonal | Cat: A19027 |
| Rabbit anti-Alix | Proteintech | Cat: 12422-1-AP |
| Rabbit anti-Tsg101 | Abclonal | Cat: A1692 |
| Rabbit anti-Bcl-2 | Abclonal | Cat: A0208 |
| Rabbit anti-Cytochrome c | Proteintech | Cat: 10993-1-AP |
| Rabbit anti-Caspase-3 | CST | Cat: 9662 |
| Rabbit anti-Cleaved Caspase-3 | CST | Cat: 9661 |
| Rabbit anti-Caspase 9/p35/p10 | Proteintech | Cat: 10380-1-AP |
| Rabbit anti-Cleaved-Caspase-9 p35 | Abbkine | Cat: ABP50010 |
| Mouse anti-Caspase 8 | CST | Cat: 9746 |
| Rabbit anti-Cleaved Caspase-8 | CST | Cat: 98134 |
| Rabbit anti-Beta Tubulin | Proteintech | Cat: 10094-1-AP |
| Rabbit anti-His | MBL | Cat: PM032 |
| Rabbit anti-EEA1 | Abcam | Cat: ab109110 |
| Mouse anti-CD63 | Invitrogen | Cat: MA1-19281 |
| Rabbit anti-LAMP1 | Abcam | Cat: ab24170 |
| Rabbit anti-Clathrin | Proteintech | Cat: 10852-1-AP |
| Goat anti-mouse IgG HRP | MultiSciences | Cat: 70-GAM007 |
| Goat anti-rabbit IgG HRP | MultiSciences | Cat: 70-GAR007 |
| Ultra-LEAF™ Purified anti-human CD63 | BioLegend | Cat: 353039 |
| Biotin anti-human CD8a | BioLegend | Cat: 372908 |
| Biotin anti-human CD4 | BioLegend | Cat: 300504 |
| 488 Conjugated Goat anti-rabbit IgG | HuaBio | Cat: HA1121 |
| 488 Conjugated Goat anti-mouse IgG | HuaBio | Cat: HA1125 |
| **Transfection Reagents** |  |  |
| JetPEI | Polyplus | Cat: 101-10N |
| Polybrene | HanBio | Cat: HB-PB-500 |
| INTERFERin^@^ | Polyplus | Cat: 409-10 |
| Other Reagents |  |  |
| Cytochrome C | SolarBio | Cat: 9007-43-6 5 |
| LY294002 | MCE | Cat: HY-10108 |
| Chlorpromazine | MCE | Cat: HY-12708 |
| Indomethacin | MCE | Cat: HY-14397 |
| N-SMase Spiroepoxide Inhibitor | Santa Cruz | Cat: 282108-77-4 |
| Doxorubicin | MCE | Cat: 23214-92-8 |
| Human CD7 Protein, His Tag | ACROBio | Cat: H52H7 |
| Blasticidin S | HanBio | Cat: HB-BSD-500 |
| Puromycin | BBI | Cat: A606719-0050 |
| 4-μm Aldehyde/Sulfate latex beads | Invitrogen | Cat: 1736853 |
| IC Fixation Buffer | Thermo | Cat: FB001 |
| Permeabilization Buffer | Thermo | Cat: 88-17000-210 |
| Triton X-100 | SolarBio | Cat: T8200 |
| Luciferin,In Vivo Grade | Promega | Cat: P1041 |
| incomplete Freund's adjuvant | Sigma–Aldrich | Cat: 32160405 |
| M.Tuberculosis Des. H37 Ra | BD | Cat: 231141 |
| BCA Protein Assay Kit | Thermo | Cat: 23225 |
| TNF alpha Mouse ELISA Kit | Thermo | Cat: 88-7324-22 |
| IL-6 Mouse ELISA Kit | Thermo | Cat: 88-7064-88 |
| IL-2 Mouse ELISA Kit | Thermo | Cat: 88-7024-88 |
| IFN gamma Mouse ELISA Kit | Thermo | Cat: 88-7314-88 |
| IgG (Total) Mouse ELISA Kit | Thermo | Cat: 88-50400-22 |
| QuantiCyto® Mouse IgM ELISA kit | Neobioscience | Cat: EMC129.48 |
| Human Biotin Positive Selection Kit | STEMCELL | Cat: 17663 |
| Cell Counting Kit-8 assay | TransGen | Cat: FC101-01 |
| iFluor™ 488 phalloidin iFluor™ | Yeasen | Cat: 40736ES75 |
| PKH26 Red Fluorescent Cell Linker Kit | Sigma-Aldrich | Cat: PKH26PCL |
| TrackVivo 680 NIR Fluorescent Imaging Agent | Fluorescence | Cat: 680-1mg |
| Antifade Mounting Medium with DAPI | Vectashield | Cat: H-1200-10 |
| Alexa Fluor™ 680 protein labeling Kit | Thermo | Cat: A20172 |
| TUNEL Detection Kit | Beyotime | Cat: C1089 |
| BUN Test Kit (Urease method) | Nanjing Jiancheng | Cat: C013-2-1 |
| AST/GOT Test Kit (Microplate method) | Nanjing Jiancheng | Cat: C010-2-1 |
| ALT/GPT Test Kit (Lai's method) | Nanjing Jiancheng | Cat: C009-2-1 |
| SCR Assay Kit (Sarcosine Oxidase Method) | Nanjing Jiancheng | Cat: C011-2-1 |
| Plasmids/Virus |  |  |
| pLVX-CMV-ABCB1(human)-FLAG-PGK-Puro | Miaoling Biology | Cat: P23940 |
| pLVX-CD7(human)-Puro | Biofeng | Cat: NM006137-16 |
| HBLV-luc-BSD | HanBio | Cat: lv53031221 |
| Anti-CD7 scFv sequence |  |  |
| V_H_ | EVQLVESGGGLVKPGGSLKLSCAASGLTFSSYAMSWVRQTPEKRLEWVA  SISSGGFTYYPDSVKGRFTISRDNARNILYLQMSSLRSEDTAMYYCARD  EVRGYLDVWGAGTTVTVSS | |
| V_L_ | AAYKDIQMTQTTSSLSASLGDRVTISCSASQGISNYLNWYQQKPDGTVK  LLIYYTSSLHSGVPSRFSGSGSGTDYSLTISNLEPEDIATYYCQQYSKL  PYTFGGGTKLEIKR | |
| siRNA |  |  |
| NC siRNA: | 5’-UUCUCCGAACGUGUCACGUdTdT-3’ | |
| *Bcl2* siRNA: | 5’-GUGAUGAAGUACAUCCAUUAUdTdT-3’ | |

Further information and requests for resources and reagents should be directed to and will be fulfilled by the Lead Contact, Zhijian Cai ([caizj@zju.edu.cn](mailto:caizj@zju.edu.cn)).
